# Combining paratransgenesis with SIT: impact of ionizing radiation on the DNA copy number of *Sodalis glossinidius in tsetse flies*

**DOI:** 10.1186/s12866-018-1283-8

**Published:** 2018-11-23

**Authors:** Güler Demirbas-Uzel, Linda De Vooght, Andrew G. Parker, Marc J. B. Vreysen, Robert L. Mach, Jan Van Den Abbeele, Adly M. M. Abd-Alla

**Affiliations:** 1 Insect Pest Control Laboratory, Joint FAO/IAEA Division of Nuclear Techniques in Food and Agriculture, Vienna International Centre, P.O. Box 100, 1400 Vienna, Austria; 2 Institute of Chemical, Environmental, and Biological Engineering, Research Area Biochemical Technology, Vienna University of Technology, Gumpendorfer Straße 1a, 1060 Vienna, Austria; 3 Department of Biomedical Sciences, Unit of Veterinary Protozoology, Institute of Tropical Medicine Antwerp (ITM), Antwerp, Belgium

**Keywords:** Symbiont, *Glossinidae*, Trypanosomosis, Trypanosomiasis, *Glossina morsitans morsitans*

## Abstract

**Background:**

Tsetse flies (Diptera: Glossinidae) are the cyclical vectors of the causative agents of African Trypanosomosis, which has been identified as a neglected tropical disease in both humans and animals in many regions of sub-Saharan Africa. The sterile insect technique (SIT) has shown to be a powerful method to manage tsetse fly populations when used in the frame of an area-wide integrated pest management (AW-IPM) program. To date, the release of sterile males to manage tsetse fly populations has only been implemented in areas to reduce transmission of animal African Trypanosomosis (AAT). The implementation of the SIT in areas with Human African Trypanosomosis (HAT) would require additional measures to eliminate the potential risk associated with the release of sterile males that require blood meals to survive and hence, might contribute to disease transmission. Paratransgenesis offers the potential to develop tsetse flies that are refractory to trypanosome infection by modifying their associated bacteria (*Sodalis glossinidius*) here after referred to as *Sodalis*. Here we assessed the feasibility of combining the paratransgenesis approach with SIT by analyzing the impact of ionizing radiation on the copy number of *Sodalis* and the vectorial capacity of sterilized tsetse males.

**Results:**

Adult *Glossina morsitans morsitans* that emerged from puparia irradiated on day 22 post larviposition did not show a significant decline in *Sodalis* copy number as compared with non-irradiated flies. Conversely, the *Sodalis* copy number was significantly reduced in adults that emerged from puparia irradiated on day 29 post larviposition and in adults irradiated on day 7 post emergence. Moreover, irradiating 22-day old puparia reduced the copy number of *Wolbachia* and *Wigglesworthia* in emerged adults as compared with non-irradiated controls, but the radiation treatment had no significant impact on the vectorial competence of the flies.

**Conclusion:**

Although the radiation treatment significantly reduced the copy number of some tsetse fly symbionts, the copy number of *Sodalis* recovered with time in flies irradiated as 22-day old puparia. This recovery offers the opportunity to combine a paratransgenesis approach – using modified *Sodalis* to produce males refractory to trypanosome infection – with the release of sterile males to minimize the risk of disease transmission, especially in HAT endemic areas. Moreover, irradiation did not increase the vector competence of the flies for trypanosomes.

**Electronic supplementary material:**

The online version of this article (10.1186/s12866-018-1283-8) contains supplementary material, which is available to authorized users.

## Background

Tsetse flies (*Glossina* spp., Diptera: *Glossinidae*) are the cyclical vectors of African trypanosomes, which cause a devastating and economically important infectious disease; sleeping sickness or Human African Trypanosomosis (HAT) in humans and nagana or Animal African Trypanosomosis (AAT) in livestock. Nagana causes high mortality in livestock and HAT is a serious health burden and risk to 60 million people in endemic regions of 36 countries in sub-Saharan Africa [1, 2]. *Trypanosoma vivax*, *T. congolense* and *T. brucei brucei* are the major tsetse transmitted pathogens in livestock [3] while *T. brucei rhodesiense* and *T. brucei gambiense* cause sleeping sickness in humans [4]. Members of the morsitans and palpalis groups of *Glossina* are efficient vectors of HAT and AAT [5]. In the absence of vaccines and efficient, safe and inexpensive drugs [6, 7], combined with increasing resistance against the current trypanocidal drugs [6, 8], control of the insect vector remains an essential part of managing disease transmission [9–11]. Most of the vector control strategies are insecticide-based [12, 13].

The sterile insect technique (SIT) is a species-specific, safe, efficient, environment friendly, biological-based control tactic to manage populations (suppression or/and elimination) of insect pests and disease vectors [14]. The SIT entails mass-rearing the target insects, sterilization of the males using ionizing radiation and sequential area-wide release of a large number of sterile males into the target area. The sterile flies compete for mating with the female wild population, interrupting their reproductive potential ultimately resulting in population reduction or elimination [15, 16].

It is crucial that when large numbers of male vectors are released their ability to transmit pathogens should be curtailed to the maximum possible extent. In past and current tsetse fly programs that had an SIT component, sterile males received two blood meals supplemented with the trypanocidal drug isometamidum chloride (10 μg/ml) before their release to minimize the risk of disease transmission. This treatment blocks the transmission ability of flies for *T. b. brucei* and reduces the transmission ability of flies for *T. congolense* by 5-fold under laboratory conditions [17]. However, a field study demonstrated that the use of this treatment was not entirely sufficient to prevent sterile males of *Glossina palpalis gambiensis* from transmitting the trypanosomes *T. congolense* and *T. vivax* [18]. Therefore, the development of tsetse fly strains refractory to trypanosome transmission would significantly contribute to the applicability of the SIT for the management of tsetse flies, especially in HAT endemic areas.

Tsetse flies harbor four main symbiotic microbes; *Wigglesworthia, Sodalis, Wolbachia* [19] and the recently found *Spiroplasma* [20]. The primary mutualist *Wigglesworthia* resides intracellularly in mycetocytes within the mycetome as well as extracellularly within maternal milk gland secretions. It provides dietary supplements that are necessary for host fecundity and is involved in the maturation process of the adult immune system [21]. In the absence of *Wigglesworthia* in the larvae, subsequent adults are characterized by an underdeveloped cellular immune system and exhibit unusual susceptibility for trypanosome infections and are sterile [21–25]. The facultative symbiont *Sodalis* displays a wide tissue tropism and is present both intra- and extracellularly in the tsetse fly midgut, muscle, fat body, milk glands, and salivary glands. The functional role of *Sodalis* in tsetse flies is relatively unknown although its influence on host longevity and modulation of susceptibility to trypanosome infection has been reported [26–28]. While all individuals in laboratory colonies harbor *Sodalis*, infection in natural populations varies in different species analyzed [29, 30]. The third symbiont, *Wolbachia* is an alpha-proteobacterium, located intracellularly in tsetse germ line tissues and is involved in cytoplasmic incompatibility. *Wolbachia* can be found in natural populations of tsetse flies with a prevalence varying between 0 and 100% depending on the species [31, 32]. Finally, *Spiroplasma* is a genus of wall-free motile, gram-positive bacteria [33, 34] associated both intracellularly and extracellularly in a variety of arthropods. It was recently identified as a novel symbiont of *G. f. fuscipes* and *G. tachinoides* [20].

Symbiotic microbes in tsetse flies have a vital role due to their significant influence on the biology of the fly, its reproduction, immunity, elicitation of phenotypes and potential effects on their vector competence for trypanosomes [35–38]. Understanding the interactions of the symbionts and parasites occuring in tsetse hosts might facilitate the development of tsetse flies refractory to trypanosome infection by modifying their symbionts. Paratransgenesis is a new genetic method based on modifying symbiotic organisms of insect vectors using recombinant technologies to express effector molecules, including ones that can potentially block pathogen development [39, 40]. As trypanosomes develop in the midgut, proventriculus and salivary glands of tsetse flies, foreign gene products need to be expressed in at least one of those tissues [23, 41]. *Sodalis* is an ideal candidate for paratransgenesis due to its presence in the midgut and the fact that it is one of the few insect bacterial symbionts that can be cultured and genetically modified in vitro [5, 42–44]. *Sodalis* has been genetically engineered to express and release significant amounts of functional anti-trypanosome nanobodies in different tissues of the tsetse fly [45]. A crucial step in implementing paratransgenesis in tsetse flies for use in SIT programs is the stable colonization of sterile male flies with recombinant *Sodalis* strains expressing trypanosome-interfering proteins. However, the impact of ionizing radiation on tsetse symbionts, especially *Sodalis*, is unknown.

The recent demonstration of tsetse pupae sex separation using near infrared imaging several days before adult emergence from the puparium [46] opens the possibility of irradiating males in the puparial stage. We investigated the impact of ionizing radiation treatment conducted at three different life stages on *Sodalis* copy number in adult *G. m. morsitans* flies at different times post emergence. Although the tsetse fly males are the sex of interest for SIT programmes, the impact of radiation on *Sodalis* copy number in females was also investigated as this effect has not been analyzed previously. After determining the optimum development phase for irradiation, i.e. having the least effect on *Sodalis* copy number, we tested the impact of irradiation on the male’s vector competence for trypanosomes as well as the impact on *Wigglesworthia,* and *Wolbachia*. We discuss the significance of our findings in the context of improving the application of SIT and paratransgenesis to manage tsetse fly populations and hence to control African trypanosomosis.

## Methods

### Tsetse fly

The colony of the tsetse fly *G. m. morsitans* used in this study originated from Zimbabwe and has been maintained at the Insect Pest Control Laboratory (IPCL) of the Joint FAO/IAEA Division of Nuclear Techniques in Food and Agriculture, Seibersdorf, Austria since 1997. The colony and experimental flies were maintained at 24 ± 0.5 °C and 75–80% RH and were fed on defibrinated bovine blood (Svaman spol s.r.o., Majava, Slovakia) using the artificial (in vitro) membrane feeding system for 15–20 min three times per week [47, 48].

### Analysis of the dynamics of *Sodalis* copy number in a *G. m. morsitans* colony

To assess the dynamics of *Sodalis* copy number in the *G. m. morsitans* colony established under laboratory rearing condition, samples of 4 males and 4 females were taken on day 0, 1, 2, 3, 4, 5, 6, 7, 14, 21 and 30 post emergence. Samples were placed at − 20 °C until DNA extraction.

### Experimental design

The impact of gamma irradiation was determined on both females and males. They were irradiated at three developmental stages to assess the effect on the copy number of *Sodalis*, *Wigglesworthia* and *Wolbachia*: (a) 7-day old adults, (b) 29-day old puparia (36 ± 12 h before emergence), (c) 22-day old puparia. Teneral tsetse males that emerged from puparia irradiated with 110 Gy on day 22 post larviposition were tested for vector competence for trypanosomes.

### Irradiation procedures

The tsetse puparia and adults were irradiated in air at the IPCL, Seibersdorf, Austria using a ^60^Co Gammacell® 220 (MDS Nordion Ltd., Ottawa, Canada). The dose rate was measured by alanine dosimetry as 2.144 Gy·sec^− 1^ on 2015-03-03 with an expanded uncertainty (k = 2) of 3.2%. The radiation field was mapped using Gafchromic HD-V2 film and the dose uniformity ratio in the volume used for the experiments was < 1.2 for adult exposures and < 1.1 for pupal exposures. The desired radiation doses were given by varying the time of exposure of the samples to give minimum doses of 20, 50 and 110 Gy (the dose currently used in tsetse SIT programs). Untreated puparia or flies were used as control (0 Gy) and handled in the same manner. For adults, 7-day old flies (males and females) were placed in small cages (11 cm diameter × 4.5 cm height) and placed in the center of the chamber for treatment. The pupae were placed in plastic Petri-dishes (diameter 5.5 cm, height 1.5 cm) that allowed irradiation in the center of the chamber.

In the first part, 7-day old males and females that had already been maintained under the above-mentioned colony conditions and offered three normal blood meals were irradiated at 48 h post the last blood meal with 20, 50 and 110 Gy at a density of 72 flies per cage with two replicates. After irradiation, all emerged flies of the different treatments and the control groups were held under standard insect rearing conditions and were offered normal blood meals every other day of the week during the length of the experiment. Four females and 4 males were frozen for each dose on day 0, 1, 7 and 14 post-irradiation. For the day 0 group, both females and males were frozen approximately 20 min after irradiation. All frozen samples were kept at − 20 °C until being used for further analysis. The experiment was replicated twice.

In the second and third part of the study, batches of puparia were collected on the same day on day 22- and 29 post larviposition. Collected puparia for each radiation dose were kept in Petri dishes and exposed to 20, 50 and 110 Gy. The experiment was replicated two and three times for 22- and 29-day old puparia, respectively. Irradiated and non-irradiated pupae were kept under standard colony conditions. Daily examinations were made for fly emergence, and non-emerged puparia were observed for each treatment. Emerged flies were collected daily and transferred to standard fly holding cages (20 cm diameter × 5 cm height) at a density of 72 flies per cage. Emerged female and male flies were held in separate holding cages during the experiment. Four females and 4 males were frozen on day 0, 1, 3, 5, 7 and 14 post emergence separately for each dose and kept at − 20 °C until further analysis.

### DNA extraction and quantitative PCR

The total DNA of each individual fly was extracted from the collected flies using the DNeasy tissue kit (QIAGEN Inc., Valencia, CA) following the manufacturer’s instructions. The extracted DNA was eluted in 200 μl elution buffer and DNA extracts from individual samples were pooled (4 females and 4 males, separately). The pooled DNA concentration was measured by spectrophotometry (Nanodrop-Synergy H1 Multi-Mode Reader, BioTek, Instruments, Inc., USA). All DNA samples were diluted to a final concentration of 4 ng/μl and 5 μl of the diluted DNA was used for qPCR to determine symbiont DNA copy number as previously described [49, 50]. The tsetse reference gene *β-tubulin* was used to normalize the qPCR reactions [50]. *Sodalis, Wigglesworthia* and *Wolbachia* densities were quantified for both sexes at different days post irradiation/emergence for all treatments by qPCR using primers that target the *fliC, codhoc* and *Wolbachia 16S rRNA* genes, respectively. The primers and the PCR conditions are given in Additional file 1.

### Tsetse fly infection with trypanosomes, maintenance, and dissection

For the infection experiment, teneral flies emerged from 22-day old irradiated (110 Gy) and non-irradiated puparia (collected and irradiated at the IPCL and shipped to the Unit of Veterinary Protozoology, Institute of Tropical Medicine (ITM), Antwerp, Belgium) were offered a blood meal containing a highly transmissible pleiomorphic *T. brucei brucei* (*Tbb*) AnTAR1 strain, 24 h after emergence. *Tbb* AnTAR1 is a post-tsetse fly strain derived from the EATRO 1125 stabilate that was originally isolated from a bushbuck in Uganda in 1966 [51]. Parasitized blood was harvested with heparin from cyclophosphamide-immune suppressed mice (Endoxan®, Baxter) 6 days post-infection and mixed with defibrinated horse blood (E&O Laboratories) to obtain > 10^6^ bloodstream form (BSF) trypanosomes/ml with 80% intermediate/stumpy forms in the infectious blood meal. This tsetse-trypanosome infection model has given good infection rates in the midgut and salivary glands of tsetse flies [52]. Flies that did not take the infectious blood meal were excluded from the experiment. Subsequently, the remaining flies were maintained for 4 weeks at 26 ± 0.5 °C and 65 ± 5% relative humidity and offered uninfected defibrinated horse blood three times per week using an artificial membrane feeding system [47]. Twenty eight days after the infective blood meal, individual flies were analyzed for the presence of procyclic and metacyclic trypanosomes (the reproductive and transmissible forms) by microscopical examination of their midguts and salivary glands, respectively. Differences in infection rates between irradiated and control flies were compared using Fisher’s exact test (two-sided) and considered significant if *P*-values were lower than 0.05.

### Statistical analysis

The statistical analysis and graphics were executed in R [53] using RStudio version 3.4.1. [54] with the packages ggplot2 v2.2.1 [55], lattice v0.20–35 [56] and MASS v7.3.47 [57]. Data was checked for normality and transformed where necessary using the Box-Cox routine. The data was log transformed where the 95% confidence interval of lambda includes 0 and transformed with (x^λ^-1)/λ in other cases. The significance of the overall differences between the different doses obtained from the various treatments was assessed by ANOVA [58]. The significance of differences between the group means (different radiation doses vs. unirradiated individually analyzed for each day post emergence and irradiation time) was determined by Tukey’s honestly significant difference (HSD) test. The *P*-values were calculated from the data with the significance threshold selected as 0.05 (Additional file 2). All regression analyses were conducted using the linear model for different times and different doses and coefficient factors (slope), t and *P* values are presented for females and males in Additional files 3 and 4 respectively.

## Results

### Dynamics of *Sodalis* copy number in non-irradiated *G. m. morsitans* adults

**Fig. 1:**
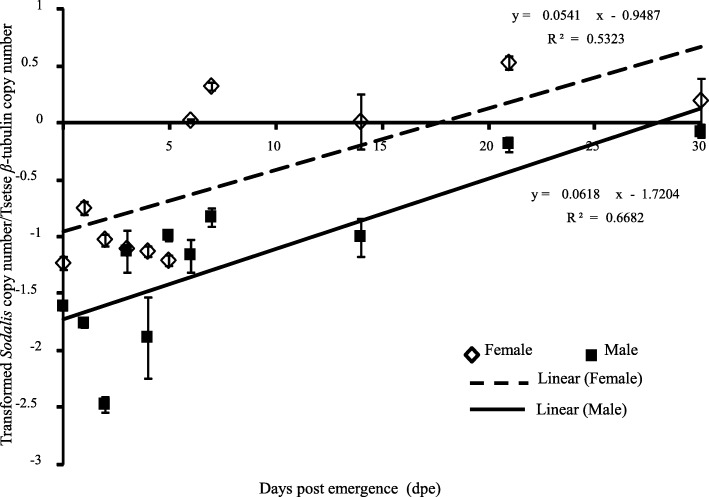
Dynamics of *Sodalis* copy number in *G. m. morsitans* adult flies maintained under laboratory colony conditions. Four males and four females were tested at each time point to estimate the *Sodalis* copy number using qPCR. Normalized qPCR data were transformed (λ = − 0.2) to best fit the normal distribution. * shows a significant difference between treatments at different levels (Tukey HSD at the 95% family wise confidence level), (* (*P* < 0.05 level), ** (*P* < 0.001), *** (*P* < 0.0001))

Experiments carried out under laboratory conditions indicated that *Sodalis* copy number was correlated with fly age and sex*.* For both males and females an exponential increase in *Sodalis* copy number was observed after fly emergence from the puparia, reaching a stable high copy number plateau when flies were aged beyond 3 weeks. In addition, *Sodalis* copy number was significantly higher in female than male flies (*P* < 0.001, regardless of fly age (Fig. 1, Additional file 2).

### Impact of irradiation on *Sodalis* copy number in *G. m. morsitans*

We evaluated the impact of irradiation on the copy number of *Sodalis* in adult flies after treatment at three different life stages: (i) as 7-day old adults, (ii) 29-day old puparia and (iii) as 22-day old puparia. Where flies were irradiated as puparia, the analyses were conducted on different days post emergence (dpe), but for flies irradiated as adults the analysis was done on different days post irradiation (dpi).

#### Adults irradiated at 7-days

**Fig. 2:**
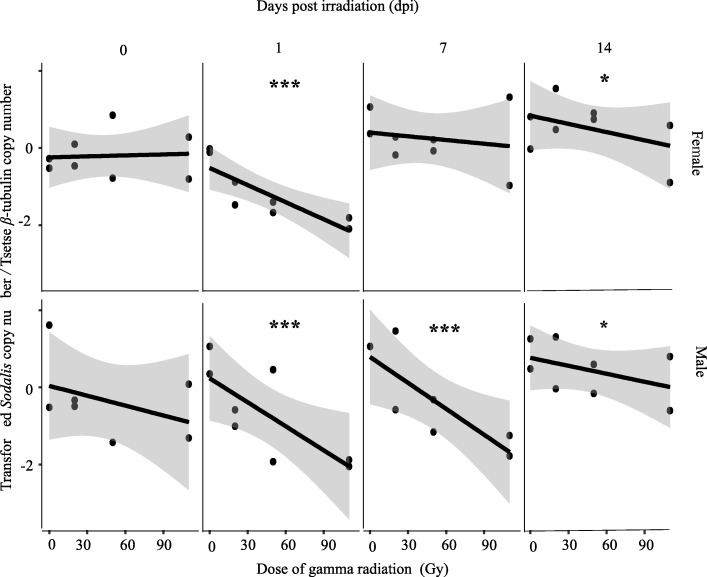
Impact of different ionizing radiation doses on *Sodalis* copy number in *G. m. morsitans* adult flies irradiated at 7-days post emergence at different times post irradiation. Four male and four female 7-day old adults exposed to different radiation doses were used to quantify *Sodalis* copy number at different time points post-irradiation. Normalized qPCR data were transformed (λ = 0.2) to best fit the statistical normal distribution. * indicates a significant different between treatments at different levels (Tukey HSD at the 95% family wise confidence level), (* (*P* < 0.05 level), ** (*P* < 0.001), *** (*P* < 0.0001))

In male flies, the radiation dose and time after irradiation significantly influenced the *Sodalis* copy number. *Sodalis* copy number decreased significantly with increasing radiation dose (*P* < 0.001), but increased significantly (*P* < 0.001, Fig. 2, Additional file 2) with time after irradiation. The negative correlation between radiation dose and *Sodalis* copy number was most obvious on day 1 and 7 post irradiation. On the day of emergence, no significant impact was observed between the different doses and *Sodalis* copy number. On day 14 post irradiation, the difference in *Sodalis* copy number among the different doses was lower than that observed on day 1 and 7 post irradiation but it remained significant (Additional file 3). For the irradiated samples and regardless of the dose, the copy number of *Sodalis* on day 14 post irradiation was relatively higher than the copy number observed on day 0, 1 and 7 day post irradiation (Fig. 2, Additional file 3). The rate of increase of *Sodalis* copy number was higher in irradiated samples than non-irradiated controls. In non-irradiated flies, there was no significant regression between *Sodalis* copy number and time (Additional file 2, Additional file 5A).

The negative impact of radiation dose on *Sodalis* copy number was lower in females than males; this was mainly obvious on day 7 post irradiation (Fig. 2, Additional file 2). The impact on the *Sodalis* population following irradiation was most obvious on day 1 post irradiation. This decrease in *Sodalis* copy number was less obvious but significant on day 14 post irradiation. No significant decrease in *Sodalis* copy number due to the increase in the dose was observed on day 7 post irradiation. On the day of irradiation, no significant regression between dose and *Sodalis* copy number was observed (Additional file 3). Over time, there was an increase in *Sodalis* copy number regardless of the dose. The increase in *Sodalis* copy number was greater in samples irradiated with 20 and 50 Gy than in 110 Gy and non-irradiated samples (Additional file 4, Additional file 5B).

#### Adults emerged from 29-day old irradiated puparia

**Fig. 3:**
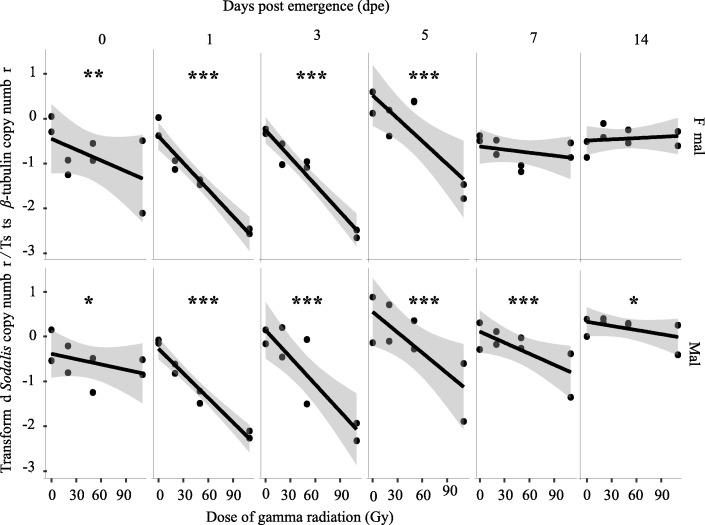
Impact of ionizing radiation on *Sodalis* copy number in *G. m. morsitans* adult flies emerged from irradiated 29-day old puparia. Four male and four female adults emerged from puparia exposed to different radiation doses at 29-days old were used to quantify *Sodalis* copy number at different time points post-emergence. Normalized qPCR data were transformed (λ = 0.2) to best fit the statistical normal distribution. * indicates a significant different between treatments at different levels (Tukey HSD at the 95% family wise confidence level), (* (*P* < 0.05 level), ** (*P* < 0.001), *** (*P* < 0.0001))

The impact of irradiation of 29-day old puparia on the *Sodalis* population was analyzed at different time-points over the course of a 14-day observation period. The irradiation significantly reduced *Sodalis* copy number in males (*P* < 0.001) (Fig. 3) irrespective of the day after emergence; however, *Sodalis* copy number significantly increased with time after emergence during the test period regardless of the dose (*P* < 0.001) (Fig. 3, Additional file 2). *Sodalis* copy number was inversely correlated with the radiation dose and was most obvious on days 1, 3 and 5 post emergence (Fig. 3, Additional file 3). Although *Sodalis* copy number was lower in irradiated males than control flies regardless of time, the increase in *Sodalis* copy number over time was higher in irradiated samples compared to control. The rate of increase in *Sodalis* copy number was higher in samples treated with 50 and 110 Gy as compared with 20 Gy. In non-irradiated samples, *Sodalis* copy number did not increase with time (Additional file 4, Additional file 6A).

In females, *Sodalis* copy number decreased significantly with increasing irradiation dose, on days 1, 3, and 5 post emergence. *Sodalis* copy number was negatively correlated with radiation dose on days 0, 1, 3, 5, 7 and 14 post emergence (Fig. 3, Additional file 3). Although the copy number of *Sodalis* in irradiated treatments was in general lower than the non-irradiated control as observed in males, an exception was found at day 14 post emergence, where *Sodalis* copy number was slightly higher than the control. In the irradiated samples, the lowest *Sodalis* copy number was found in the samples treated with 110 Gy except on day 7 post emergence, where the lowest copy number was observed in 50 Gy-irradiated samples. As in males, there was a significant positive regression between *Sodalis* copy number and time post emergence (*P* < 0.01) in the female samples irradiated at 110 Gy (Fig. 2B, Additional file 4). The rate of increase in *Sodalis* copy number was higher in the 110 Gy irradiated samples as compared with that in 20 and 50 Gy irradiated samples. Surprisingly a significant decrease in *Sodalis* copy number over time was observed in non-irradiated samples (*P* = 0.011) (Additional file 4, Additional file 6B).

#### Adults emerged from 22-day old irradiated pupae

**Fig. 4:**
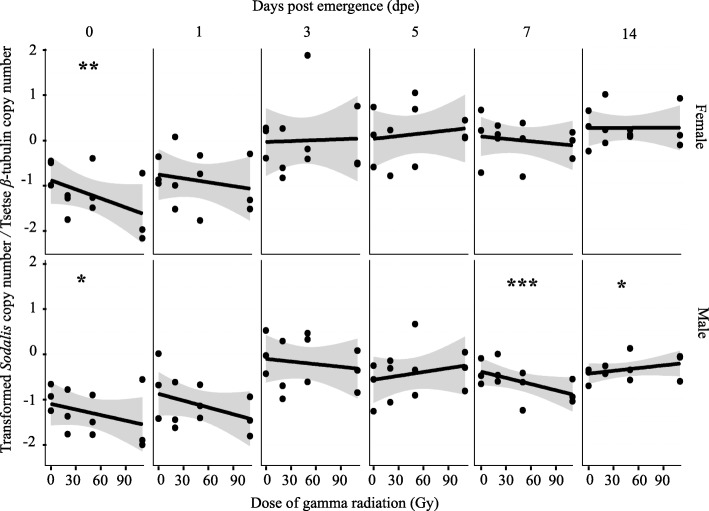
Impact of ionizing radiation on *Sodalis* copy number in *G. m. morsitans* adult flies emerged from irradiated 22-day old puparia. Four male and four female adults emerged from puparia exposed to different radiation doses at 22-days old were used to quantify *Sodalis* copy number at different time point post-emergence. Normalized qPCR data were transformed (λ = 0.26) to best fit the statistical normal distribution. * indicates a significant different between treatment at different levels (Tukey HSD at the 95% family wise confidence level), (* (*P* < 0.05 level), ** (*P* < 0.001), *** (*P* < 0.0001))

The quantification of *Sodalis* copy number in adult flies (males and females) emerged from puparia irradiated at 22-days old showed a different profile from that observed in flies irradiated as adults or as 29-day old puparia. However, day post emergence and sex significantly affected *Sodalis* copy number whilst *Sodalis* copy number in general was independent of radiation dose (Fig. 4). As there was a significant interaction between time and treatment (*P* = 0.017) and between sex and time (*P* < 0.01) and treatment, the data for each time were analyzed separately for males and females (Additional file 2).

In male flies, *Sodalis* copy number was in general not affected by dose, but was significantly affected by day post emergence when all data was analyzed together (Fig. 4, Additional file 2). However, when analyzed on each day post emergence, increasing doses induced a decrease in *Sodalis* copy number on day 0, 1, 3 and 7 post emergence. In contrast, *Sodalis* copy number increased with increasing radiation dose on day 5 and 14 post emergence (Fig. 4, Additional file 3). The rate of increase of *Sodalis* copy number in non-irradiated controls was not significant with time but was significant for the 20, 50 and 110 Gy-treatment groups (Additional file 4, Additional file 7A).

In general, the copy number of *Sodalis* was higher in female than in male flies and was independent of radiation dose, but increased significantly with time (Fig. 4, Additional file 2. However, the rate of increase of *Sodalis* copy number with time in the samples irradiated with 20 and 110 Gy was much higher than the rate of increase in the samples irradiated with 50 Gy and non-irradiated controls (Additional file 2, Additional file 7B).

### Impact of tsetse developmental stage during irradiation on *Sodalis* copy number in *G. m. morsitans* males

**Fig. 5:**
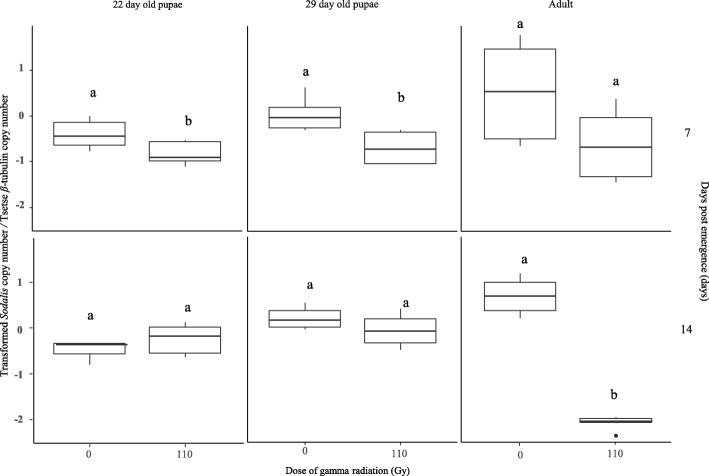
Impact of tsetse *G. m. morsitans* developmental stage during irradiation with 110 Gy on *Sodalis* copy number in *G. m. morsitans* males. Different letter show significant differences

Comparing the *Sodalis* copy number in the non-irradiated control with that in males irradiated with 110 Gy on day 22 and 29 post larviposition and adults, indicated that on day 7 post emergence, the *Sodalis* copy number was significantly lower than in non-irradiated males (*P* = 0.002), irrespective of the developmental stage at the time of irradiation. On day 14 post emergence, the copy number of *Sodalis* in males irradiated with 110 Gy as adult males was significantly lower than non-irradiated males (*P* < 0.001). *Sodalis* copy number was not significantly different in males emerged from puparia irradiated on day 29 and day 22 post larviposition as compared with non-irradiated control flies. It is worth noting that the highest and lowest copy number of *Sodalis* was observed in males in the irradiated adult treatment in controls and 110 Gy respectively (Fig. 5).

### Impact of irradiation of 22-day old puparia on *Wigglesworthia* and *Wolbachia* copy number in *G. m. morsitans* flies

**Fig. 6:**
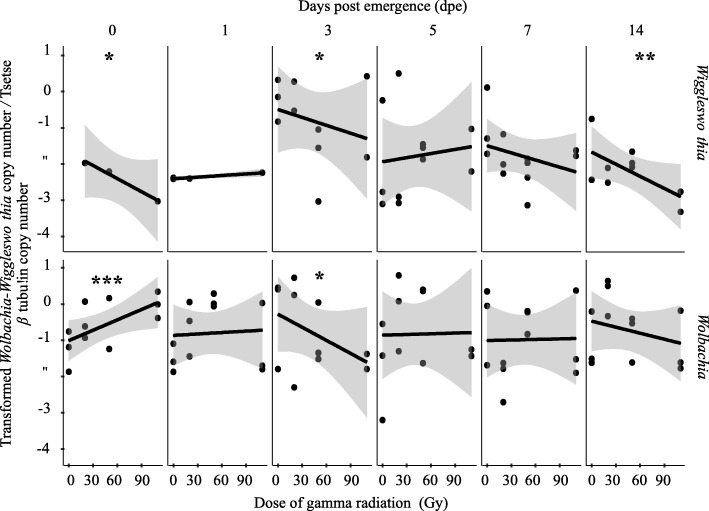
Impact of ionizing radiation on *Wigglesworthia and Wolbachia* copy number in *G. m. morsitans* males. Four male and four female adults emerged from puparia exposed to different radiation doses at 22-days old were used to quantify *Wigglesworthia and Wolbachia* copy number at different time points post-emergence. Normalized qPCR data were transformed λ = 0.02 and λ = 0.2) to best fit the statistical normal distribution. * indicates a significant different between treatments at different levels (Tukey HSD at the 95% family wise confidence level), (* (*P* < 0.05 level), ** (*P* < 0.001), *** (*P* < 0.0001))

*Wigglesworthia* and *Wolbachia* densities were significantly different in female flies as compared with male flies (Fig. 6 and Additional file 8). In addition, there was a significant interaction between sex and treatment in *Wolbachia*; therefore, the data for males and females were analyzed separately (Additional file 2).

In males, the ANOVA indicated that increasing irradiation dose and time did not cause significant changes in *Wigglesworthia* copy number when analyzed separately (Fig. 6, Additional file 2). However, the regression analysis indicated that the copy number of *Wigglesworthia* was reduced with increasing dose regardless of the time post emergence but this negative regression was only significant on days 0, 3 and 14 post emergence (Additional file 3). It is important to note that unlike *Sodalis*, the *Wigglesworthia* copy number did not significantly change with time in non-irradiated males or males irradiated with 50 Gy. In males irradiated with 20 and 110 Gy the *Wigglesworthia* copy number decreased significantly with time (Fig. 6, Additional file 4, Additional file 8A). The copy number of *Wolbachia* in male flies was not significantly affected by the radiation dose (Additional file 2). *Wolbachia* copy number increased with increasing dose on the day of emergence. This positive correlation turned into a significant negative correlation on day 3 post emergence (Fig. 6, Additional file 3). The copy number of *Wolbachia* did not change significantly over time for non-irradiated or irradiated males (Fig. 6, Additional file 2, Additional file 9A).

**Fig. 7:**
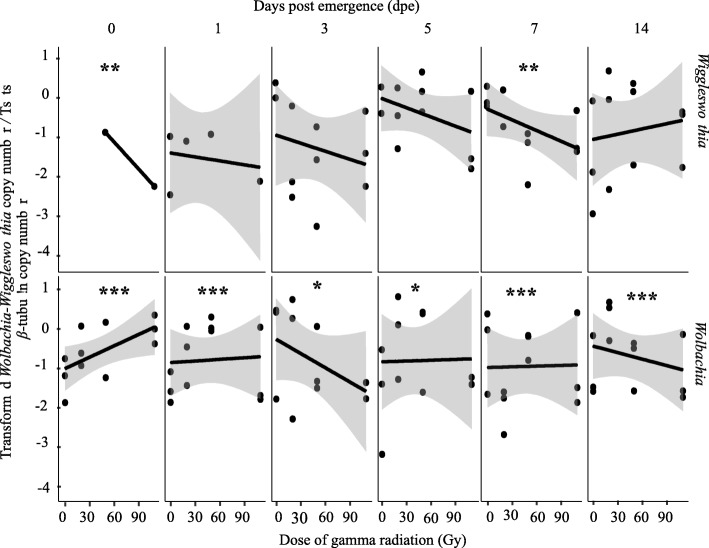
Impact of ionizing radiation on *Wigglesworthia and Wolbachia* copy number in *G. m. morsitans* females. Four males and four females of adults emerged from 22-day old puparia exposed to different radiation doses were used to quantify *Wigglesworthia and Wolbachia* copy number at different time point post-emergence. Normalized qPCR data were transformed (λ = 0.3 and λ = − 0.04) to best fit the statistical normal distribution. * indicates a significant different between treatments at different levels (Tukey HSD at the 95% family wise confidence level), (* (*P* < 0.05 level), ** (*P* < 0.001), *** (*P* < 0.0001))

In female flies, increasing radiation dose or time post emergence did not causes significant changes in the copy number of *Wigglesworthia* (Fig. 7, and Additional file 2). In general, the copy number of *Wigglesworthia* was reduced with increasing dose up to day 7 post emergence but seemed to increase with increasing dose on day 14 post emergence (Additional file 3). There was no significant correlation between copy number of *Wigglesworthia* and time in non-irradiated control flies, whereas significant positive correlation was observed in the 110 Gy treatment group (Additional file 4, Additional file 9A).

Unlike *Wigglesworthia,* the copy number of *Wolbachia* in female flies was significantly affected by radiation dose and time post emergence (Fig. 7**,** Additional file 2). In general, similar to *Wigglesworthia* the copy number of *Wolbachia* decreased with increasing doses, with the exception on the day of emergence when the copy number of *Wolbachia* increased. The decrease in *Wolbachia* copy number was significant on days 1, 3, 5, 7 and 14 post emergence (Additional file 3). Over time, the *Wolbachia* copy number increased significantly only in 20 Gy treated females (Additional file 4, Additional file 9B).

### Impact of irradiation on the tsetse fly’s susceptibility towards trypanosomes

Following per os challenge with trypanosomes in their 1st blood meal, 14.7% and 6% of non-irradiated and irradiated *G. m. morsitans* adults, respectively developed a mature trypanosome infection in their salivary glands (Table 1). At the midgut level a similar infection ratio was observed, i.e. 18% and 6% in non-irradiated and irradiated flies, respectively. However, the observed differences in infection rates between irradiated and non-irradiated flies in both the salivary gland and the midgut were not statistically significant (Table 1). Results from this experiment show that the establishment of a trypanosome infection in the tsetse’s midgut and the subsequent maturation of this infection were not significantly affected by irradiation.

**Table 1:** Light Microscope^a^ evaluation of the proportion of male irradiated and non-irradiated control *G. m. morsitans* flies infected with *T. b. brucei*

Treatment	Infected/ total # flies		Maturation	*P* values	
	Midgut glands	Salivary gland rate		Midgut glands	Salivary gland rate
Non-irradiated	11/61	9/61	0.82	–	–
Irradiated	3/50	3/50	1	0.0839	0.219

*p* values were obtained by comparing the infection prevalence of the irradiated group to the infection prevalence of non-treated control flies using a two-sided Fisher’s exact test.

^a^Light-Microscope observations/evaluations conducted using 100× magnification to carry out the observations

^b^Maturation rate: salivary gland/midgut infected flies

## Discussion

The implementation of the SIT in the context of an area-wide integrated pest management strategy was successful in eradicating a population of *Glossina austeni* from Unguja Island of Zanzibar [57]. However, the release of large numbers of sterile male flies bears a potential risk of temporarily increasing disease transmission during the initial release phase of the programme [58]. To date, the release of sterile male tsetse flies has only been implemented in areas without HAT. Before their release, the sterile males are offered blood meals mixed with an anti-trypanosomal drug (isometamidium chloride) and, although this protocol reduced the risk of increased trypanosome transmission, there are reports that claim that it does not completely prevent it [17, 18]. Therefore, the implementation of a programme with an SIT component in an HAT endemic area will require additional measures to eliminate the risk of increased trypanosome transmission.

One possibility would be to use paratransgenesis to develop tsetse flies refractory to trypanosome infection by exploiting the presense of symbiotic bacteria associated with the flies. It has been suggested to modify the symbiotic bacteria *Sodalis* to produce anti-trypanosome factors [42, 45, 52, 59] and important recent progress can be reported with the development of paratransgenic tsetse flies [52, 60, 61] for use in SIT programmes [58]. However, as the males destined for release need to be irradiated to make them sterile, the impact of the irradiation treatment on the *Sodalis* community needed to be assessed*.* Therefore, we investigated the effect of different radiation doses administered during different life stages on the copy number of *Sodalis* in *G. m. morsitans* flies.

It is known that the SIT becomes more effective when only males are released, but separating tsetse male from female puparia is currently not possible at an operational scale. In operational SIT programmes implemented so far, tsetse fly males have been separated from females using one the following methods: (i) manual separation of the adults based on the morphological differences, or (ii) exploiting the difference in pupal period (females emerge 2 days earlier than males) [62, 63]. A third method is based on the use of near infrared light [46] to separate the puparia 8–10 days before adults emergence, but this is still under development. The above mentioned methods offer opportunities to irradiate male flies as adults (method 1) or pupae (methods 2 and 3) and to sterilize them for the release in an SIT program. Hence, the importance of analysing the impact of irradiation on tsetse symbionts at these different developmental phases. The selected male separation method depends on the conditions of each SIT program: (1) in the program that successfully eradicated a population of *G. austeni* from Unguja Island of Zanzibar [57], adult males were separated manually from adult females and the males irradiated and released as adults. A similar strategy was used for the programme against *Glossina palpalis gambiensis* and *Glossina tachinoides* in Sidéradougou, Burkina Faso [64] and against *G. fuscipes fuscipes* and *G. pallidipes* in Ethiopia [65]. A different approach was adopted in the pilot SIT programme against *Glossina morsitans* in Tanzania, where the flies were irradiated and released as pupae in release stations [66]. Another approach was adopted in the program in Senegal against *G. p. gambiensis* where the male puparia were collected on day 29 post larviposition after the emergence of females, irradiated and shipped under chilled conditions at 10 °C from several countries to Dakar, Senegal [67]. Upon arrival, the pupae were left to emerge and the male flies were released as adults in the target area. In the latter case, it is important to point out that separating male and female puparia during the mid-pupal phase (between days 15–25 post larviposition) would be much appreciated in SIT programs as it would allow the irradiation and shipment of male puparia under ideal environmental condions (e.g. 23 °C), which would result in better quality males. With insects like the Mediterranean fruit fly *Ceratitis capitata*, the problem was solved through the development of genetic sexing strains (GSS), which allows the females to be eliminated at the embryonic or pupal stage. This approach greatly increased the efficacy of SIT programmes against this pest and significantly reduced its cost [68, 69]. Unfortunately such an approach is not available for tsetse flies.

The use of ionizing radiation to sterilize male insects is a simple process that is easy and safe to apply [70]. Radiation causes single- and double-strand breaks in the chromosomes of both somatic and germ line cells [71], resulting in the formation of dominant lethal mutations in eggs and sperm [70]. However, as a result of the irradiation free radicals originating from water radiolysis, mainly OH free radicals, H atoms and solvated electrons e_aqu_, are formed in the treated insect that interact with intra- or extracellular molecules. The free radicals affect the microbial communities associated with irradiated flies as an indirect effect of irradiation. The negative impact of irradiation on reducing the gut microbiota was previously demonstrated in humans [72], but the impact on the microbiota associated with insects was so far not reported.

The results show that the copy number of *Sodalis* in untreated male and female *G. m. morsitans* significantly increased with time. Non irradiated female *G. m. morsitans* had a higher *Sodalis* copy number than male flies during a period of 30 days after emergence. This contrasts with earlier work that showed that *Sodalis* densities in male *G. p. gambiensis* were always higher than in female flies over a period of 80 days [73], and this difference might be due to a species-specific impact on *Sodalis* copy number or the size of the analyzed samples (*n* = 8) at each time point. In general, the copy number of *Sodalis* infection in somatic tissues increased with the age of the fly but varied with species and sex [23]. In addition, our results indicate that the *Sodalis* population was significantly reduced after irradiation of 7-day old adult males, with no significant recovery on day 14 post irradiation. In contrast, the recovery of *Sodalis* copy number was significant in adult flies treated as 22 or 29 day-old puparia. The recovery in *Sodalis* copy number was most prominent in female flies when treated as 29-day old puparia, and in male flies when treated as 22-day old puparia. The observed recovery in *Sodalis* copy number in adult flies treated as pupae might be due to the relative longer period available for multiplication of *Sodalis* individuals after irradiation in comparison with the shorter period available in irradiated adult males. It is important to note that *Sodalis* has a relatively slow growth rate (~ 15 h for cell population doubling times in vitro) and therefore a relatively longer period is needed to increase its copy number in the irradiated host [59].

**Fig. 8:**
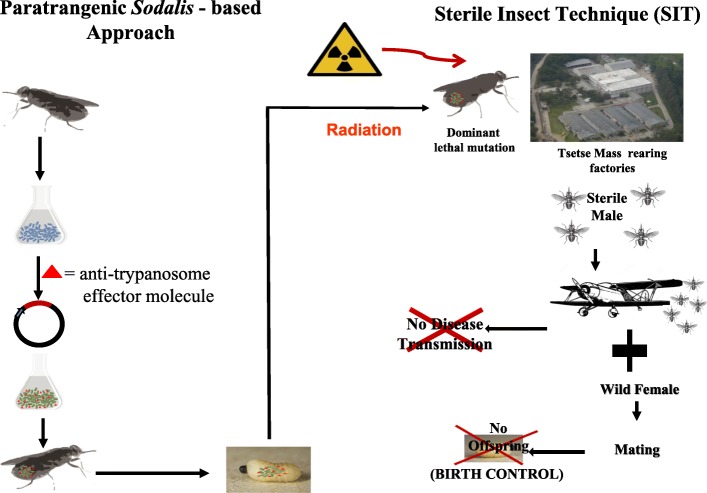
Schematic diagram of the combination between paratransgenesis and sterile insect technique (SIT). The proposed strategy to reduce and/or eliminate risk of increasing trypanosome transmission that might be associated with the release of large numbers of tsetse males during tsetse SIT implementation programs

The recovery of *Sodalis* copy number in males treated as 22 day-old puparia to similar or even higher levels as observed in non-irradiated males opens the opportunity to use paratransgenesis to develop tsetse strains that are refractory to trypanosome infection. Although this study was conducted on non-modified *Sodalis*, it can at this stage be assumed that the response of modified *Sodalis* to irradiation would be similar to wild *Sodalis*, but this will need to be confirmed by further research. In our study both puparia and adult flies were irradiated to estimate the optimal dose and effects on *Sodalis* copy number, and the results clearly indicate that irradiating adult flies prohibits the use of paratransgenesis to develop tsetse strains that are refractory to trypanosome infection. Therefore, the most effective use of paratransgenesis in SIT programs will be achieved when separating the male from the female puparia on day 22 post larviposition using near infrared light, at least for *G. m. morsitans* (Fig. 8) [46]. This method, however, is still under development and it is important to note that the successful development and use of paratransgenesis in SIT programs might be species dependent and is most certainly closely linked to an optimization of male and female pupal separation protocols.

The results also indicate a general reduction in the copy number of *Wigglesworthia* and *Wolbachia* in irradiated flies, especially when the dose was a high as 110 Gy. Whereas *Wigglesworthia* has a clear role in tsetse females as it provides vitamins necessary for female fertility [78], the role of *Wigglesworthia* in males is not clear and therefore we cannot speculate on the impact of a reduction in *Wigglesworthia* copy number in irradiated males. On the other hand, the reduction in *Wolbachia* copy number might negatively affect the potential of releasing of *Wolbachia* infected males to enhance sterile insect applications using the cytoplasmic incompatibility (CI) phenomenon as recenly implemented in mosquitoes [31, 37, 74–76]. The reduction in *Wolbachia* copy number after gamma radiation treatment was previously reported in *Brugia malayi* adult worms [77]. On the other hand, an enhancement effect on *Sodalis*, *Wigglesworthia* and *Wolbachia* densities was observed with lower radiation doses.

Tsetse flies are in general refractory to parasite transmission as illustrated by the extremely low natural prevalence of trypanosome-infected tsetse flies (< 0.1% for *T. brucei*) as well as by the low tsetse fly infection rates that are obtained in optimal experimental laboratory conditions. In the vector competence experiment, the results from adults treated as puparia on day 22 post larviposition show that the establishment of a trypanosome infection in the tsetse’s midgut and the subsequent maturation of this infection was not affected by the irradiation. However, the pending research question will be to determine effect of irradiation on the vectorial capacity of adult tsetse flies infected with genetically modified *Sodalis* expressing trypanosome-interfering molecules.

To date, no previous study has been conducted to assess the effect of ionizing radiation on the tsetse flies’ symbiont copy number. This study determined the impact of irradiating puparia and adults of *G. m. morsitans* on the copy number of *Sodalis, Wigglesworthia* and *Wolbachia*. Our data indicate that irradiation does not affect the vectorial capacity of the released sterile males, and hence, measures are needed to address this problem. The data of this study are encouraging for the use of paratransgensis to develop strains that are refractory to trypanosome infection, which will reduce or eliminate any potential risk that might be associated with the release of sterile males in HAT endemic areas.

## Conclusion

This study provides the first demonstration of the functional impact of irradiation on *Sodalis glossinidus* and the vectorial capacity of treated flies. When puparia are irradiated between day 22 and 29 post larviposition, a significant recovery in *Sodalis* copy number occurs in the adult flies, but the vectorial capacity of adult males is not affected. Moreover, irradiaton induces a significant reduction in the copy number of *Wigglesworthia* and *Wolbachia*. The current study also reinforces the idea for the potential use of *Sodalis* to be developed into a paratransgenic platform that can be combined with SIT to block transmission of trypanosomes.

## Additional files

Additional file 1: List of Primers used for quantitative PCR (qPCR) analyses of microbiome in *Glossina* species (DOCX 27 kb)

Additional file 2: ANOVA Statistics for Interaction (DOCX 20 kb)

Additional file 3 Regression Statistics for different time post irradiation (DOCX 28 kb)

Additional file 4: Regression Statistics for different irradiation doses. (DOCX 25 kb)

Additional file 5: Impact of time post irradiation on *Sodalis* copy number in *G. m. morsitans* adult flies irradiated at 7-day post emergence. Four males (A) and four females (B) of 7-day old adults exposed to different radiation doses were used to quantify *Sodalis* copy number at different time point post-irradiation foe each irradiation dose. Normalized qPCR data were transformed (λ = 0.2) to best fit the statistical normal distribution and used for the regression analysis. (PDF 89 kb)

Additional file 6: Impact of time post irradiation on *Sodalis* copy number in *G. m. morsitans* adult flies emerged from irradiated 29-day old puparia. Four males (A) and four females (B) of adults emerged from 29-day old puparia exposed to different radiation doses were used to quantify *Sodalis* copy number at different time point post-irradiation foe each irradiation dose. Normalized qPCR data were transformed ((λ = 0.2) to best fit the statistical normal distribution and used for the regression analysis. (PDF 95 kb)

Additional file 7: Impact of time post irradiation on *Sodalis* copy number in *G. m. morsitans* adult flies emerged from irradiated 22-day old puparia. Four males (A) and four females (B) of adults emerged from 22-day old puparia exposed to different radiation doses were used to quantify *Sodalis* copy number at different time point post-irradiation foe each irradiation dose. Normalized qPCR data were transformed (λ = 0.26) to best fit the statistical normal distribution and used for the regression analysis. (PDF 103 kb)

Additional file 8: Impact of time post irradiation on *Wigglesworthia and Wolbachia* copy number in *G. m. morsitans* adult flies emerged from irradiated 22-day old puparia. Four males of adults emerged from 22-day old puparia exposed to different radiation doses were used to quantify *on Wigglesworthia male* (A) and *Wigglesworthia* female (B) copy number at different time point post-irradiation for each irradiation dose. Normalized qPCR data were transformed (λ = 0.02 and λ = 0.3 for males and females respectively) to best fit the statistical normal distribution and used for the regression analysis. (PDF 99 kb)

Additional file 9: Impact of time post irradiation on *Wigglesworthia and Wolbachia* copy number in *G. m. morsitans* adult flies emerged from irradiated 22-day old puparia. Four females of adults emerged from 22-day old puparia exposed to different radiation doses were used to quantify *on Wolbachia* male (A) and *Wolbachia* female (B) copy number at different time point post-irradiation for each irradiation dose. Normalized qPCR data were transformed ((λ = 0.2 and λ = − 0.04 for males and females respectively) to best fit the statistical normal distribution and used for the regression analysis. (PDF 127 kb)

## Abbreviations

AAT: Animal African Trypanosomosis
AW-IPM: Area-wide integrated pest management programs
DNA: Deoxyribonucleic acid
dpe: Days post emergence
dpi: Days post irradiation
GSS: Genetic sexing strains
HAT: Human African Trypanosomosis
HSD: Tukey’s honesty significant difference test
IPCL: Insect pest control laboratories
ITM: Institute of Tropical Medicine
qPCR: Quantitative polymerase chain reaction
RH: Relative humidity
SIT: Sterile insect technique
