# Supplementary material for: Combining paratransgenesis with SIT: impact of ionizing radiation on the DNA copy number of Sodalis glossinidius in tsetse flies
Source: BMC Microbiol. 2018 Nov 23;18(Suppl 1):160. doi: 10.1186/s12866-018-1283-8 (PMC6251162; doi:10.1186/s12866-018-1283-8)
Supplement: Supplementary file 1 — List of Primers used for quantitative PCR (qPCR) analyses of microbiome in Glossina species (DOCX 27 kb) [file 12866_2018_1283_MOESM1_ESM.docx]

Additional File 1. List of Primers used for quantitative PCR (qPCR) analyses of microbiome in *Glossina* species

| **Target Gene** | **Primer Name** | **Primer Sequence**  **(Listed 5- to -3)** | **Annealing Temperature (^°^C)** | **Amplicon Size (bp)** | **References** |
| --- | --- | --- | --- | --- | --- |
| fliC (flagellin) (*Sodalis*) | sodqPCR-FliCF | GAA GCC ACC GAT CCT GTA AC | 55 | 508 | [1] |
|  | sodqPCR-FliCR | CAT CTT TGC CCG TAG AAA TCA C |  |  |  |
| Codhoc (*Wigglesworthia*) | WiggqPCRcodhocF2 | GACTTGTACGTGATATTTCCAAGC | 60 | 645 | [2] |
|  | WiggqPCRcodhocR2 | GACATCAAATCGCGTTACTGG |  |  |  |
| Wolbachia 16S rRNA(*Wolbachia*) | Wsp fwd | YATACCTATTCGAAGGGATAG | 60 | 438 | [3, 4] |
|  | Woltse- cyt R | GGATTAGCTTAGCCTCGC |  |  |  |
| β-tubulin  (Tsetse Fly) | Tsetse-tubulinF | GATGGTCAAGTGCGATCCT | 55 | 355 | [5] |
|  | Tsetse-tubulinR | TGAGAACTCGCCTTCTTC C |  |  |  |

Reference List

1. Weiss BL, Maltz M, Aksoy S: Obligate symbionts activate immune system development in the tsetse fly. J Immunol 2012, 188:3395-3403.

2. Rose TM, Schultz ER, Henikoff JG, Pietrokovski S, McCallum CM, Henikoff S: Consensus-degenerate hybrid oligonucleotide primers for amplification of distantly related sequences. Nucleic Acids Res 1998, 26:1628-1635.

3. Doudoumis V, Tsiamis G, Wamwiri F, Brelsfoard C, Alam U, Aksoy E, Dalaperas S, Abd-Alla A, Ouma J, Takac P, Aksoy S, Bourtzis K: Detection and characterization of Wolbachia infections in laboratory and natural populations of different species of tsetse flies (genus Glossina). BMC Micobiology 2012, 12:S3.

4. Brelsfoard C, Tsiamis G, Falchetto M, Gomulski LM, Telleria E, Alam U, Doudoumis V, Scolari F, Benoit JB, Swain M, Takac P, Malacrida AR, Bourtzis K, Aksoy S: Presence of extensive Wolbachia symbiont insertions discovered in the genome of its host Glossina morsitans morsitans. PLoS Negl Trop Dis 2014, 8:e2728.

5. Identification of a functional Antigen5-related allergen in the saliva of a blood feeding insect, the tsetse fly. In Insect Biochem Mol Biol 2009, 39:332-341.
