# Supplementary material for: Combining paratransgenesis with SIT: impact of ionizing radiation on the DNA copy number of Sodalis glossinidius in tsetse flies
Source: BMC Microbiol. 2018 Nov 23;18(Suppl 1):160. doi: 10.1186/s12866-018-1283-8 (PMC6251162; doi:10.1186/s12866-018-1283-8)
Supplement: Supplementary file 2 — ANOVA Statistics for Interaction (DOCX 20 kb) [file 12866_2018_1283_MOESM2_ESM.docx]

Additional File 2. ANOVA Statistics for Interaction

| **Treatment** | **Interactions** | **Both Female and Male** | | | **Female** | | | **Male** | | |
| --- | --- | --- | --- | --- | --- | --- | --- | --- | --- | --- |
|  |  | **F** | **df** | **P value** | **F** | **df** | **P value** | **F** | **df** | **P value** |
| Impact of irradiation on *Sodalis* in tsetse irradiated as Adults | Sex | 0.40 | 1, 178 | 0.527867 | - | - | - | - | - | - |
|  | Time | 59.73 | 1, 178 | 7.654e-13 | 42.20 | 1, 92 | 4.146e-09 | 21.37 | 1, 86 | 1.320e-05 |
|  | Treatment | 53.11 | 1, 178 | 9.856e-12 | 9.39 | 1, 92 | 0.002 | 49.19 | 1, 86 | 4.968e-10 |
|  | Sex – Time | 0.93 | 1, 178 | 0.33 | - | - | - | - | - | - |
|  | Sex - Treatment | 9.12 | 1, 178 | 0.002888 | - | - | - | - | - | - |
|  | Time – Treatment | 0.60 | 1, 178 | 0.43 | 0.05 | 1, 92 | 0.81 | 1.39 | 1, 86 | 0.24 |
|  | Sex- Time- Treatments | 0.74 | 1, 178 | 0.38 | - | - | - | - | - | - |
| Impact of irradiation on *Sodalis* in tsetse irradiated as 29-day old pupae | Sex | 31.12 | 1, 279 | 5.721e-08 | - | - | - | - | - | - |
|  | Time | 86.74 | 1, 279 | < 2.2e-16 | 6.33 | 1, 44 | 0.01 | 25.81 | 1, 44 | 7.370e-06 |
|  | Treatment | 232.04 | 1, 279 | < 2.2e-16 | 28.49 | 1, 44 | 3.137e-06 | 40.77 | 1, 44 | 9.133e-08 |
|  | Sex – Time | 5.009 | 1, 279 | 0.02 | - | - | - | - | - | - |
|  | Sex - Treatment | 0.07 | 1, 279 | 0.79 | - | - | - | - | - | - |
|  | Time – Treatment | 37.46 | 1, 279 | 3.148e-09 | 11.60 | 1, 44 | 0.001 | 2.73 | 1, 44 | 0.1054 |
|  | Sex- Time- Treatments | 3.30 | 1, 279 | 0.07 | - | - | - | - | - | - |
| Impact of irradiation on *Sodalis* in tsetse irradiated as 22-day old pupae | Sex | 44.33 | 1, 417 | 8.743e-11 | - | - | - | - | - | - |
|  | Time | 134.20 | 1, 417 | < 2.2e-16 | 27.27 | 1, 63 | 2.116e-06 | 14.28 | 1, 68 | 0.0003 |
|  | Treatment | 4.55 | 1, 417 | 0.03 | 0.74 | 1, 63 | 0.52 | 1.11 | 1, 68 | 0.29 |
|  | Sex – Time | 9.21 | 1, 417 | 0.002546 | - | - | - | - | - | - |
|  | Sex - Treatment | 0.01 | 1, 417 | 0.90 | - | - | - | - | - | - |
|  | Time – Treatment | 5.57 | 1, 417 | 0.01 | 0.68 | 1, 63 | 0.56 | 1.51 | 1, 68 | 0.222 |
|  | Sex- Time- Treatments | 0.001 | 1, 417 | 0.97192 | - | - | - | - | - | - |
| Impact of irradiation on *Wolbachia* in tsetse irradiated as 22-day old pupae | Sex | 537.58 | 1,389 | < 2.2e-16 | - | - | - | - | - | - |
|  | Time | 1.69 | 1,389 | 0.19 | 8.47 | 1, 187 | 0.004 | 0.02 | 1, 202 | 0.86 |
|  | Treatment | 12.69 | 1,389 | 0.0004114 | 52.13 | 1, 187 | 1.268e-11 | 0.36 | 1, 202 | 0.546 |
|  | Sex – Time | 1.53 | 1,389 | 0.21 | - | - | - | - | - | - |
|  | Sex - Treatment | 20.49 | 1,389 | 7.97e-06 | - | - | - | - | - | - |
|  | Time – Treatment | 3.81 | 1,389 | 0.05 | 0.880 | 1, 187 | 0.34 | 3.21 | 1, 202 | 0.07 |
|  | Sex- Time- Treatments | 0.002 | 1,389 | 0.9595567 | - | - | - | - | - | - |
| Impact of irradiation on *Wigglesworthia* in tsetse irradiated as 22-day old pupae | Sex | 18.40 | 1, 94 | 4.339e-05 | - | - | - | - | - | - |
|  | Time | 0.02 | 1, 94 | 0.87 | 0.96 | 1, 48 | 0.32 | 1.15 | 1, 46 | 0.28 |
|  | Treatment | 3.15 | 1, 94 | 0.07 | 2.12 | 1, 48 | 0.15 | 2.23 | 1, 46 | 0.14 |
|  | Sex – Time | 2.09 | 1, 94 | 0.15 | - | - | - | - | - | - |
|  | Sex - Treatment | 0.01 | 1, 94 | 0.91 | - | - | - | - | - | - |
|  | Time – Treatment | 0.43 | 1, 94 | 0.511 | 1.64 | 1, 48 | 0.20 | 0.24 | 1, 46 | 0.62 |
|  | Sex- Time- Treatments | 1.45 | 1, 94 | 0.23 | - | - | - | - | - | - |
| Sodalis density of normal colony flies in tsetse irradiated as Adults | Sex | 36.46 | 1, 62 | 9.581e-08 |  |  |  |  |  |  |
|  | Time | 79.42 | 1, 62 | 1.053e-12 | 30.26 | 1, 31 | 5.102e-06 | 43.256 | 1, 31 | 2.404e-07 |
|  | Treatment | - | - | - | - | - | - | - | - | - |
|  | Sex – Time | 0.35 | 1, 62 | 0.5543 |  |  |  |  |  |  |
|  | Sex - Treatment | - | - | - | - | - | - |  | - | - |
|  | Time – Treatment | - | - | - | - | - | - | - | - | - |
|  | Sex- Time- Treatments | - | - | - | - | - | - | - | - | - |
