# Supplementary material for: Combining paratransgenesis with SIT: impact of ionizing radiation on the DNA copy number of Sodalis glossinidius in tsetse flies
Source: BMC Microbiol. 2018 Nov 23;18(Suppl 1):160. doi: 10.1186/s12866-018-1283-8 (PMC6251162; doi:10.1186/s12866-018-1283-8)
Supplement: Supplementary file 3 — Regression Statistics for different time post irradiation (DOCX 28 kb) [file 12866_2018_1283_MOESM3_ESM.docx]

Additional File 3. Regression Statistics for different days post irradiation

| **Treatment*** | **Days Post Emergence**** | **Female** | | | | | | | **Male** | | | | | | |
| --- | --- | --- | --- | --- | --- | --- | --- | --- | --- | --- | --- | --- | --- | --- | --- |
|  |  | **Coefficient** | **t value** | **df** | ***P* value** | **Intercept t value** | **Intercept *P* value** | **R-squared (R^2^)** | **Coefficient** | **t value** | **df** | ***P* value** | **Intercept t value** | **Intercept *P* value** | **R-squared (R^2^)** |
| Impact of irradiation on *Sodalis* in tsetse irradiated as Adults | 0 | 0.000 | 0.327 | 22 | 0.74 | -1.327 | 0.198 | -0.0404 | -0.0085 | -1.780 | 19 | 0.091 | 0.046 | 0.963 | 0.0977 |
|  | 1 | - 0.013 | -7.060 | 22 | 4.4e-07 | -4.294 | 0.0002 | 0.6799 | -0.0215 | -5.487 | 22 | 1.63e-05 | 0.952 | 0.351 | 0.5586 |
|  | 7 | -0.0028 | -0.812 | 22 | 0.42 | 1.856 | 0.07 | -0.0150 | -0.0227 | -6.038 | 19 | 8.29e-06 | 3.135 | 0.0054 | 0.6393 |
|  | 14 | -0.0071 | -2.126 | 22 | 0.04 | 4.235 | 0.0003 | 0.1327 | -0.0067 | -2.326 | 22 | 0.02 | 4.187 | 0.0003 | 0.1973 |
| Impact of irradiation on *Sodalis* in tsetse irradiated as 29-day old pupae | 0 | -0.0056 | -2.822 | 22 | 0.009 | -3.365 | 0.002 | 0.2324 | -0.0034 | -2.218 | 22 | 0.03 | -3.399 | 0.002 | 0.1455 |
|  | 1 | -0.0124 | -10.424 | 22 | 5.64e-10 | -5.627 | 1.17e-05 | 0.824 | -0.0108 | -12.072 | 22 | 3.54e-11 | -5.387 | 2.07e-05 | 0.8629 |
|  | 3 | -0.0123 | -15.987 | 22 | 1.35e-13 | -6.923 | 5.97e-07 | 0.9171 | -0.0142 | -5.949 | 22 | 5.49e-06 | 4.561 | 0.0001 | 0.5993 |
|  | 5 | -0.0139 | -5.911 | 22 | 5.99e-06 | 3.398 | 0.002 | 0.5961 | -0.01302 | -5.303 | 21 | 2.94e-05 | 3.877 | 0.0008 | 0.5521 |
|  | 7 | -0.0018 | -1.668 | 22 | 0.109 | -8.102 | 4.78e-08 | 0.0719 | -0.0067 | -4.860 | 22 | 7.39e-05 | 1.142 | 0.266 | 0.4959 |
|  | 14 | 0.0007 | 0.724 | 22 | 0.477 | -6.783 | 8.15e-07 | -0.0211 | -0.0030 | -2.291 | 22 | 0.03 | 4.286 | 0.0003 | 0.1559 |
| Impact of irradiation on *Sodalis* in tsetse irradiated as 22-day old pupae | 0 | -0.0062 | -3.161 | 34 | 0.003 | -7.184 | 2.62e-08 | 0.2044 | -0.0037 | 0.003 | 34 | 0.04 | -9.669 | 2.74e-11 | 0.0880 |
|  | 1 | -0.0028 | -1.283 | 33 | 0.208 | -5.241 | 9.06e-06 | 0.01864 | -0.0038 | 0.208 | 33 | 0.05 | -8.005 | 3.1e-09 | 0.0774 |
|  | 3 | 0.0007 | 0.241 | 33 | 0.811 | -0.122 | 0.904 | -0.0284 | -0.0020 | -0.957 | 33 | 0.34 | -0.843 | 0.405 | -0.0024 |
|  | 5 | 0.0020 | 0.896 | 33 | 0.377 | 0.256 | 0.800 | -0.0058 | 0.0030 | 1.339 | 34 | 0.18 | -4.191 | 0.0001 | 0.0221 |
|  | 7 | -0.0018 | -1.038 | 34 | 0.306 | 0.867 | 0.392 | 0.0022 | -0.0044 | -3.725 | 34 | 0.0007 | -5.163 | 1.06e-05 | 0.2689 |
|  | 14 | -0.0003 | -0.165 | 26 | 0.870 | 2.406 | 0.023 | -0.0373 | 0.0019 | 2.202 | 33 | 0.03 | -7.887 | 4.3e-09 | 0.1017 |

| Impact of irradiation on *Wigglesworthia* in tsetse irradiated as 22-day old pupae | 0 | -0.0158 | -5.920 | 4 | 0.004 | -1.137 | 0.319 | 0.8719 | -0.0157 | -3.104 | 6 | 0.021 | -5.828 | 0.001 | 0.5523 |
| --- | --- | --- | --- | --- | --- | --- | --- | --- | --- | --- | --- | --- | --- | --- | --- |
|  | 1 | -0.0051 | -1.679 | 12 | 0.119 | -6.915 | 1.62e-05 | 0.1228 | -0.0050 | -1.231 | 5 | 0.27 | -18.173 | 9.27e-06 | 0.0792 |
|  | 3 | -0.0063 | -1.608 | 28 | 0.119 | -2.853 | 0.008 | 0.0518 | -0.0109 | -2.118 | 26 | 0.04 | -0.984 | 0.3343 | 0.1144 |
|  | 5 | -0.0062 | -2.038 | 30 | 0.050 | -0.616 | 0.54 | 0.09238 | 0.0102 | 1.268 | 27 | 0.21 | -6.047 | 1.87e-06 | 0.0212 |
|  | 7 | -0.0073 | -2.853 | 29 | 0.007 | -2.106 | 0.04 | 0.1922 | -0.0061 | -1.117 | 29 | 0.27 | -6.270 | 7.62e-07 | 0.0081 |
|  | 14 | 0.0034 | 0.736 | 31 | 0.467 | -3.147 | 0.003 | -0.0145 | -0.0177 | -3.588 | 21 | 0.001 | -8.210 | 5.42e-08 | 0.3506 |
| Impact of irradiation on *Wolbachia* in tsetse irradiated as 22-day old pupae | 0 | 0.0078 | 3.976 | 31 | 0.0003 | -7.610 | 1.4e-08 | 0.3164 | 0.0078 | 3.976 | 31 | 0.0003 | -7.610 | 1.4e-08 | 0.3164 |
|  | 1 | -0.0313 | -4.171 | 30 | 0.0002 | -15.269 | 1.09e-15 | 0.3459 | 0.0012 | 0.0002 | 34 | 0.69 | -4.415 | 9.72e-05 | -0.0247 |
|  | 3 | -0.0392 | -2.397 | 31 | 0.0227 | 2.096 | 0.04 | 0.1292 | -0.0112 | -2.588 | 31 | 0.014 | -1.163 | 0.253 | 0.1512 |
|  | 5 | -0.0249 | -2.102 | 30 | 0.044 | -7.180 | 5.46e-08 | 0.0993 | -0.0004 | -0.085 | 31 | 0.932 | -2.975 | 0.005 | -0.0320 |
|  | 7 | -0.0415 | -5.659 | 32 | 2.92e-06 | -9.458 | 8.71e-11 | 0.4846 | -0.0001 | -0.035 | 34 | 0.972 | -3.907 | 0.0004 | -0.0293 |
|  | 14 | -0.0533 | -6.763 | 29 | 2.01e-07 | -8.660 | 1.55e-09 | 0.5986 | -0.0052 | -1.608 | 33 | 0.117 | -2.201 | 0.034 | 0.0445 |

*Treatment = radiation doses (0, 20, 50 and 110 Gy); **Time = 0, 1, 3, 5, 7 and 14 days post emergence (post irradiation for treated adults). Each day is representing symbiont copy number changes according to different treatments.
