# Supplementary material for: Combining paratransgenesis with SIT: impact of ionizing radiation on the DNA copy number of Sodalis glossinidius in tsetse flies
Source: BMC Microbiol. 2018 Nov 23;18(Suppl 1):160. doi: 10.1186/s12866-018-1283-8 (PMC6251162; doi:10.1186/s12866-018-1283-8)
Supplement: Supplementary file 4 — Regression Statistics for different irradiation doses. (DOCX 25 kb) [file 12866_2018_1283_MOESM4_ESM.docx]

Additional File 4. Slope values for Regression Statistics for different doses

| **Treatment** | **DOSE*** | **Female** | | | | | | | **Male** | | | | | | |
| --- | --- | --- | --- | --- | --- | --- | --- | --- | --- | --- | --- | --- | --- | --- | --- |
|  |  | **Coefficient** | **t value** | **df** | ***P* value** | **Intercept t value** | **Intercept *P* value** | **R-squared (R^2^)** | **Coefficient** | **t value** | **df** | ***P* value** | **Intercept t value** | **Intercept *P* value** | **R-squared (R^2^)** |
| Impact of irradiation on *Sodalis* in tsetse irradiated as Adults | 0 Gy | 0.056 | 3.185 | 22 | 0.004 | -0.973 | 0.34131 | 0.284 | 0.020 | 0.849 | 19 | 0.406 | 3.194 | 0.004 | -0.014 |
|  | 20 Gy | 0.120 | 5.678 | 22 | 1.04e-05 | -4.242 | 0.000334 | 0.575 | 0.094 | 3.37 | 22 | 0.002 | -2.59 | 0.016 | 0.310 |
|  | 50 Gy | 0.107 | 3.944 | 22 | 0.000691 | -3.286 | 0.003372 | 0.387 | 0.091 | 2.867 | 19 | 0.009 | -4.339 | 0.0003 | 0.265 |
|  | 110 Gy | 0.068 | 1.807 | 22 | 0.08 | -2.888 | 0.00854 | 0.089 | 0.089 | 2.748 | 22 | 0.011 | -6.022 | 4.63e-06 | 0.221 |
| Impact of irradiation on *Sodalis* in tsetse irradiated as 29-day old pupae | 0 Gy | -0.035 | -2.686 | 34 | 0.011 | -0.207 | 0.8372 | 0.150 | 0.024 | 1.754 | 34 | 0.088 | -0.540 | 0.592 | 0.05598 |
|  | 20 Gy | 0.047 | 4.427 | 34 | 9.37e-05 | -11.014 | 9.30e-13 | 0.347 | 0.064 | 4.760 | 33 | 3.74e-05 | -4.256 | 0.0001 | 0.389 |
|  | 50 Gy | 0.033 | 1.841 | 34 | 0.07 | -6.101 | 6.36e-07 | 0.063 | 0.086 | 6.405 | 34 | 2.58e-07 | -8.465 | 6.93e-10 | 0.533 |
|  | 110 Gy | 0.077 | 5.129 | 34 | 1.17e-05 | -14.506 | 3.99e-16 | 0.419 | 0.072 | 4.782 | 34 | 3.29e-05 | -11.580 | 2.40e-13 | 0.384 |
| Impact of irradiation on *Sodalis* in tsetse irradiated as 22-day old pupae | 0 Gy | 0.062 | 4.411 | 51 | 5.33e-05 | -4.710 | 1.95e-05 | 0.262 | 0.022 | 1.392 | 52 | 0.17 | -6.051 | 1.6e-07 | 0.017 |
|  | 20 Gy | 0.115 | 7.334 | 51 | 1.62e-09 | -8.849 | 7.03e-12 | 0.503 | 0.064 | 4.631 | 52 | 2.47e-05 | -10.925 | 4.48e-15 | 0.278 |
|  | 50 Gy | 0.071 | 2.912 | 51 | 0.005 | -3.275 | 0.00190 | 0.125 | 0.055 | 2.927 | 50 | 0.00514 | -7.173 | 3.22e-09 | 0.1292 |
|  | 110 Gy | 0.121 | 5.965 | 51 | 2.33e-07 | -7.300 | 1.83e-09 | 0.399 | 0.075 | 4.637 | 51 | 2.49e-05 | -10.271 | 5.15e-14 | 0.282 |
| Impact of irradiation on *Wigglesworthia* in tsetse irradiated as 22-day old pupae | 0 Gy | -0.060 | -1.863 | 32 | 0.07 | -0.677 | 0.5035 | 0.069 | -0.017 | -0.249 | 34 | 0.804 | -3.115 | 0.003 | -0.02753 |
|  | 20 Gy | 0.063 | 1.675 | 31 | 0.10 | -3.868 | 0.000526 | 0.053 | -0.163 | -2.264 | 28 | 0.031 | -2.088 | 0.046 | 0.1246 |
|  | 50 Gy | 0.046 | 1.591 | 36 | 0.12 | -4.615 | 4.84e-05 | 0.039 | -0.019 | -0.515 | 32 | 0.610 | -7.792 | 6.9e-09 | -0.022 |
|  | 110 Gy | 0.070 | 2.725 | 39 | 0.009 | -8.026 | 8.75e-10 | 0.138 | -0.129 | -1.934 | 24 | 0.064 | -3.578 | 0.001 | 0.098 |
| Impact of irradiation on *Wolbachia* in tsetse irradiated as 22-day old pupae | 0 Gy | 0.116 | 1.605 | 52 | 0.115 | -8.679 | 1.09e-11 | 0.028 | 0.017 | 0.63 | 51 | 0.531 | -5.81 | 4.07e-07 | -0.011 |
|  | 20 Gy | 0.317 | 4.262 | 51 | 8.76e-05 | -14.837 | < 2e-16 | 0.248 | 0.034 | 1.219 | 52 | 0.228 | -3.941 | 0.0002 | 0.009 |
|  | 50 Gy | -0.033 | -0.41 | 45 | 0.68 | -11.43 | 6.74e-15 | -0.018 | -0.037 | -1.79 | 49 | 0.079 | -1.92 | 0.060 | 0.042 |
|  | 110 Gy | 0.109 | 0.781 | 35 | 0.44 | -9.871 | 1.19e-11 | -0.010 | -0.029 | -1.255 | 46 | 0.216 | -5.063 | 7.1e-06 | 0.012 |

*Dose = radiation doses (0, 20, 50 and 110 Gy); Time = 0, 1, 3, 5, 7 and 14 days post emergence (post irradiation for treated adults). Each day is representing symbiont copy number changes according to different dose.
