# Supplementary figures and images for: Combining paratransgenesis with SIT: impact of ionizing radiation on the DNA copy number of Sodalis glossinidius in tsetse flies
Source: BMC Microbiol. 2018 Nov 23;18(Suppl 1):160. doi: 10.1186/s12866-018-1283-8 (PMC6251162; doi:10.1186/s12866-018-1283-8)

**A**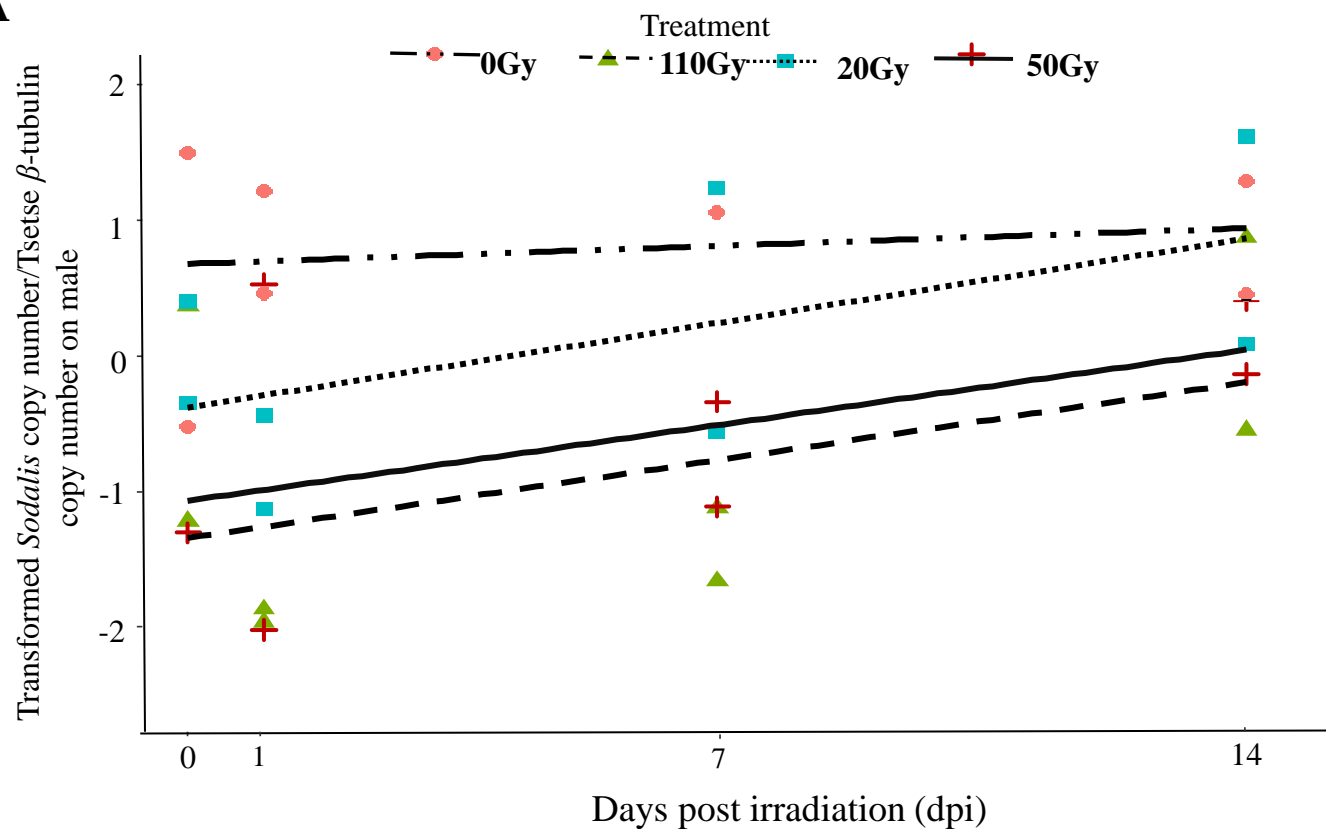**B**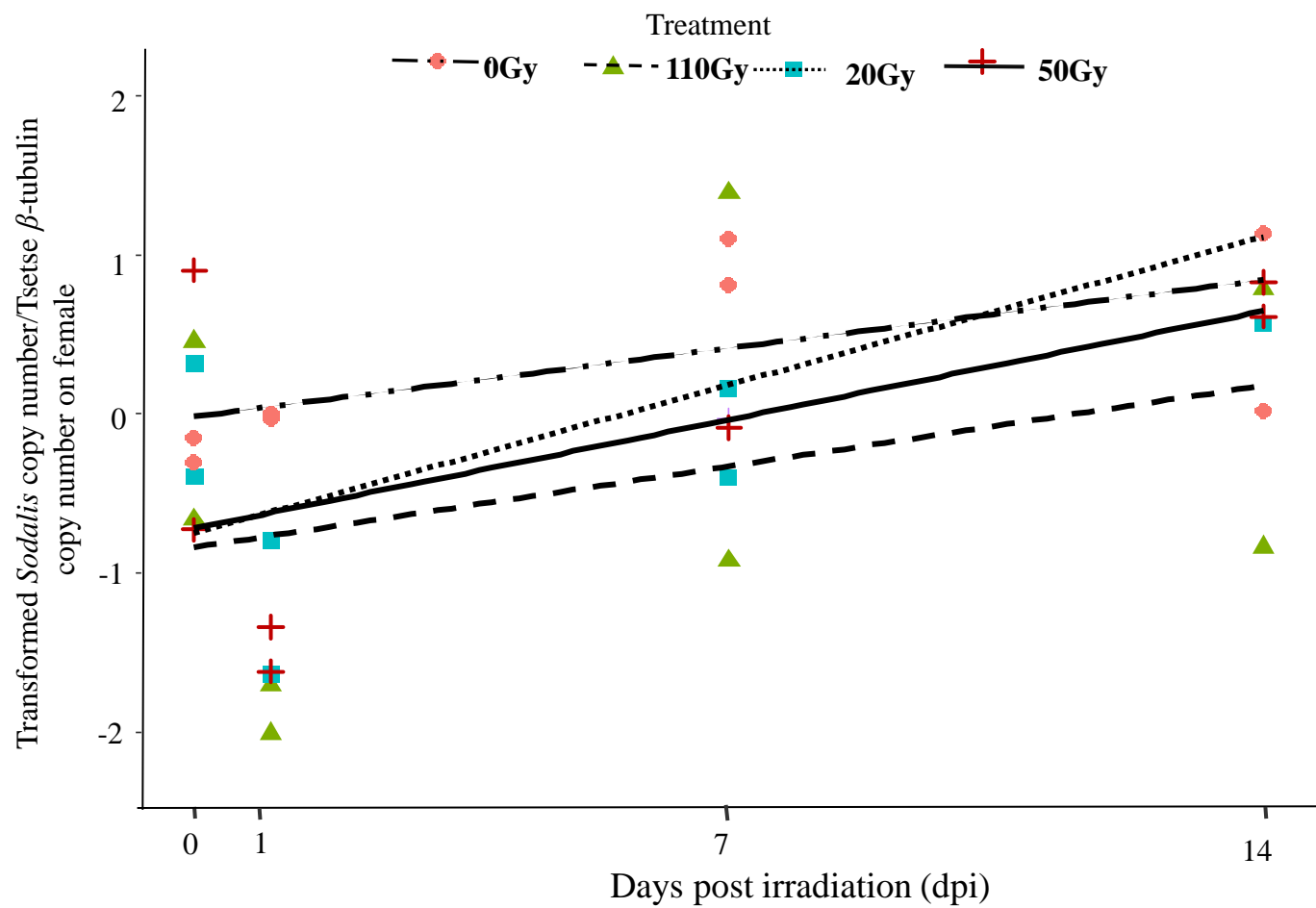

Supplement: Supplementary file 5 — Impact of time post irradiation on Sodalis copy number in G. m. morsitans adult flies irradiated at 7-day post emergence. Four males (A) and four females (B) of 7-day old adults exposed to different radiation doses were used to quantify Sodalis copy number at different time point post-irradiation foe each irradiation dose. Normalized qPCR data were transformed (λ = 0.2) to best fit the statistical normal distribution and used for the regression analysis. (PDF 89 kb) [file 12866_2018_1283_MOESM5_ESM.pdf]

**A**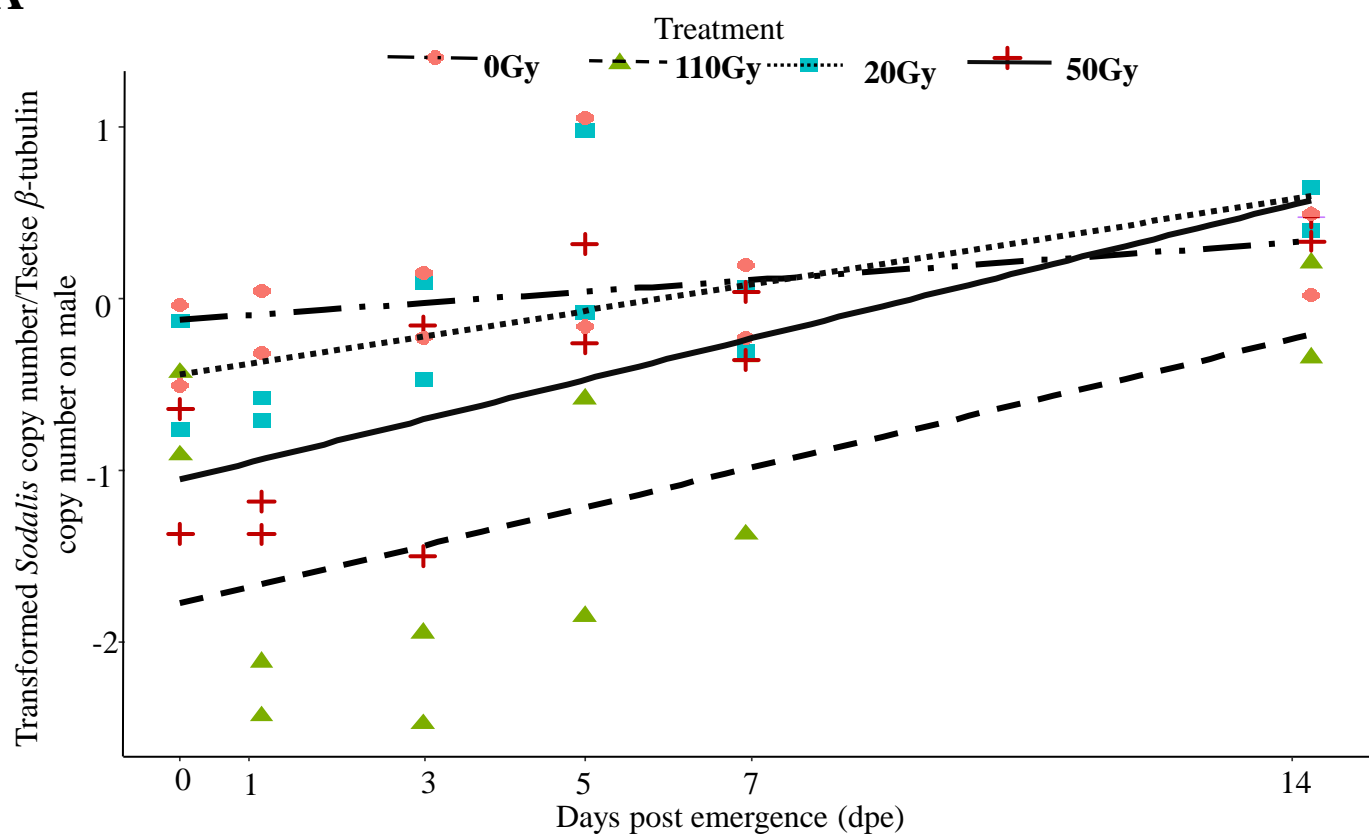**B**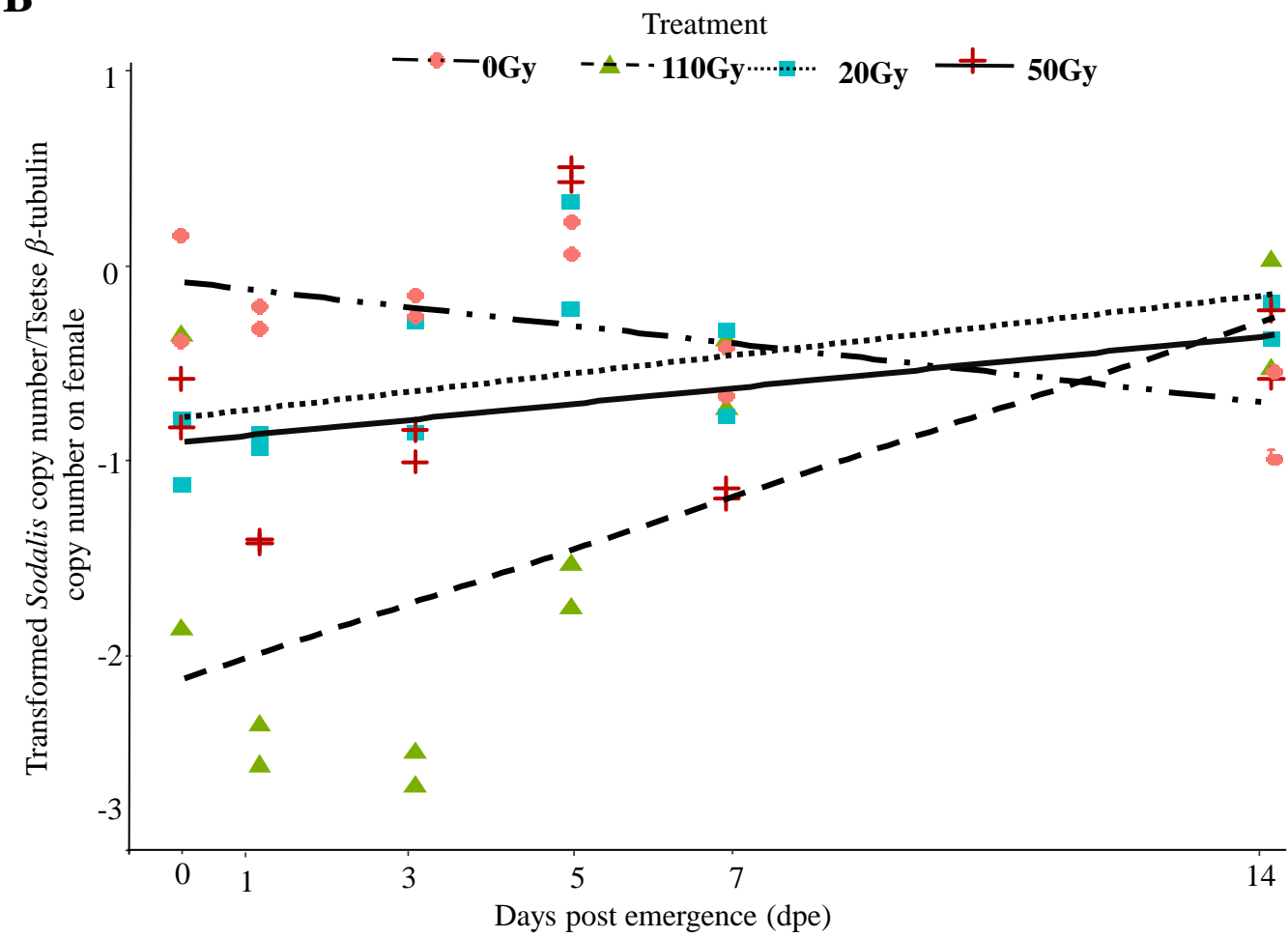

Supplement: Supplementary file 6 — Impact of time post irradiation on Sodalis copy number in G. m. morsitans adult flies emerged from irradiated 29-day old puparia. Four males (A) and four females (B) of adults emerged from 29-day old puparia exposed to different radiation doses were used to quantify Sodalis copy number at different time point post-irradiation foe each irradiation dose. Normalized qPCR data were transformed ((λ = 0.2) to best fit the statistical normal distribution and used for the regression analysis. (PDF 95 kb) [file 12866_2018_1283_MOESM6_ESM.pdf]

**A**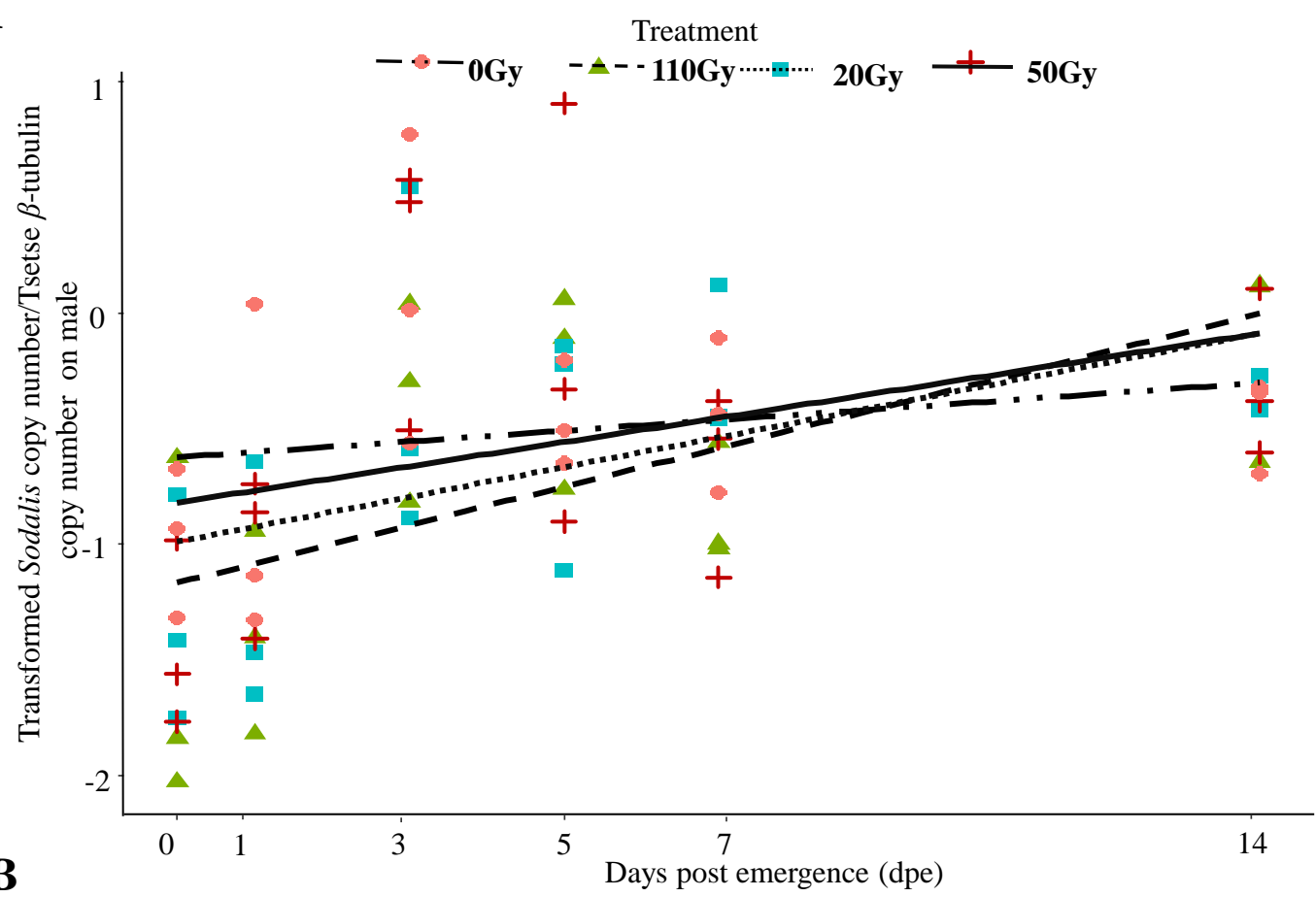**B**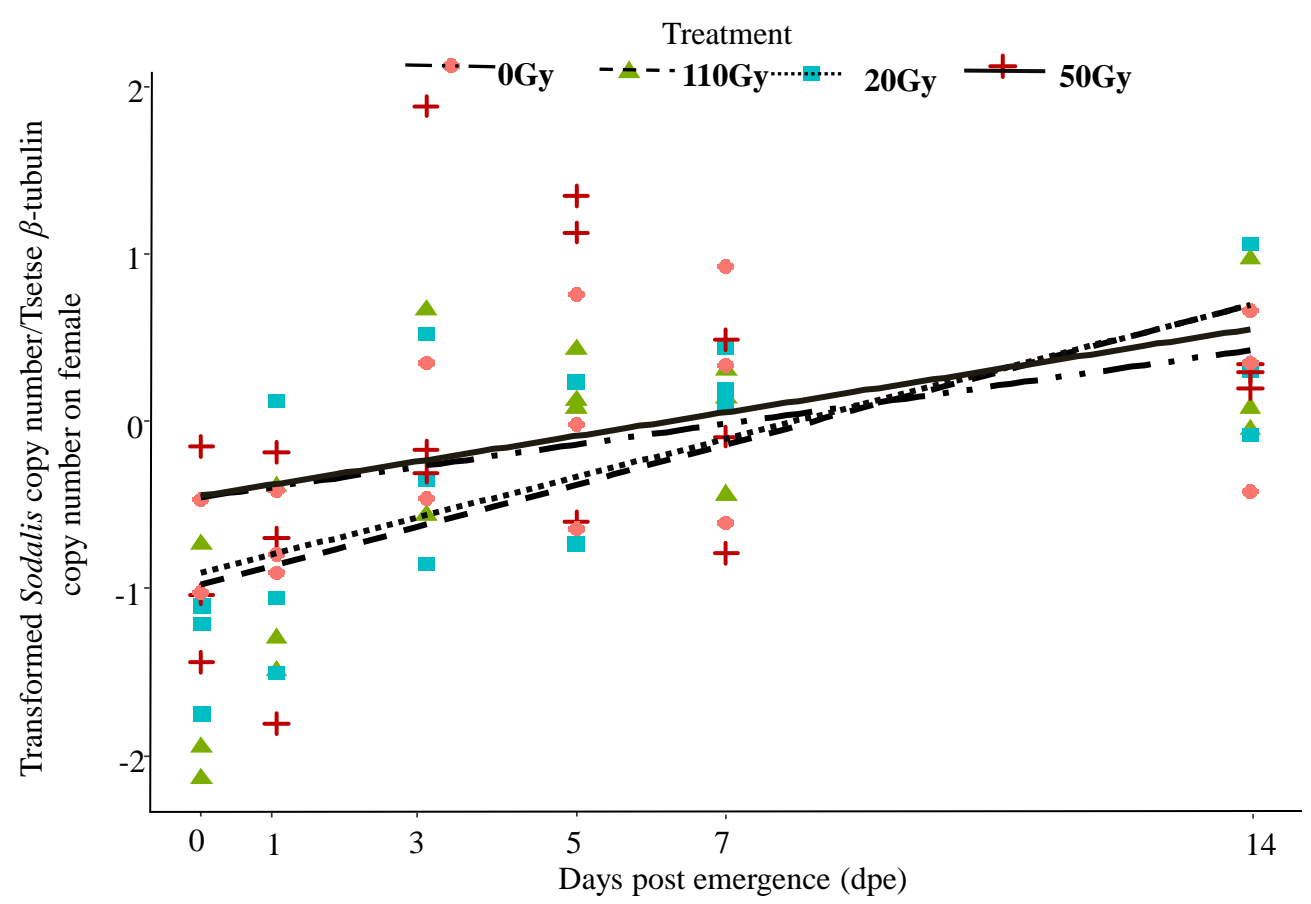

Supplement: Supplementary file 7 — Impact of time post irradiation on Sodalis copy number in G. m. morsitans adult flies emerged from irradiated 22-day old puparia. Four males (A) and four females (B) of adults emerged from 22-day old puparia exposed to different radiation doses were used to quantify Sodalis copy number at different time point post-irradiation foe each irradiation dose. Normalized qPCR data were transformed (λ = 0.26) to best fit the statistical normal distribution and used for the regression analysis. (PDF 103 kb) [file 12866_2018_1283_MOESM7_ESM.pdf]

**A**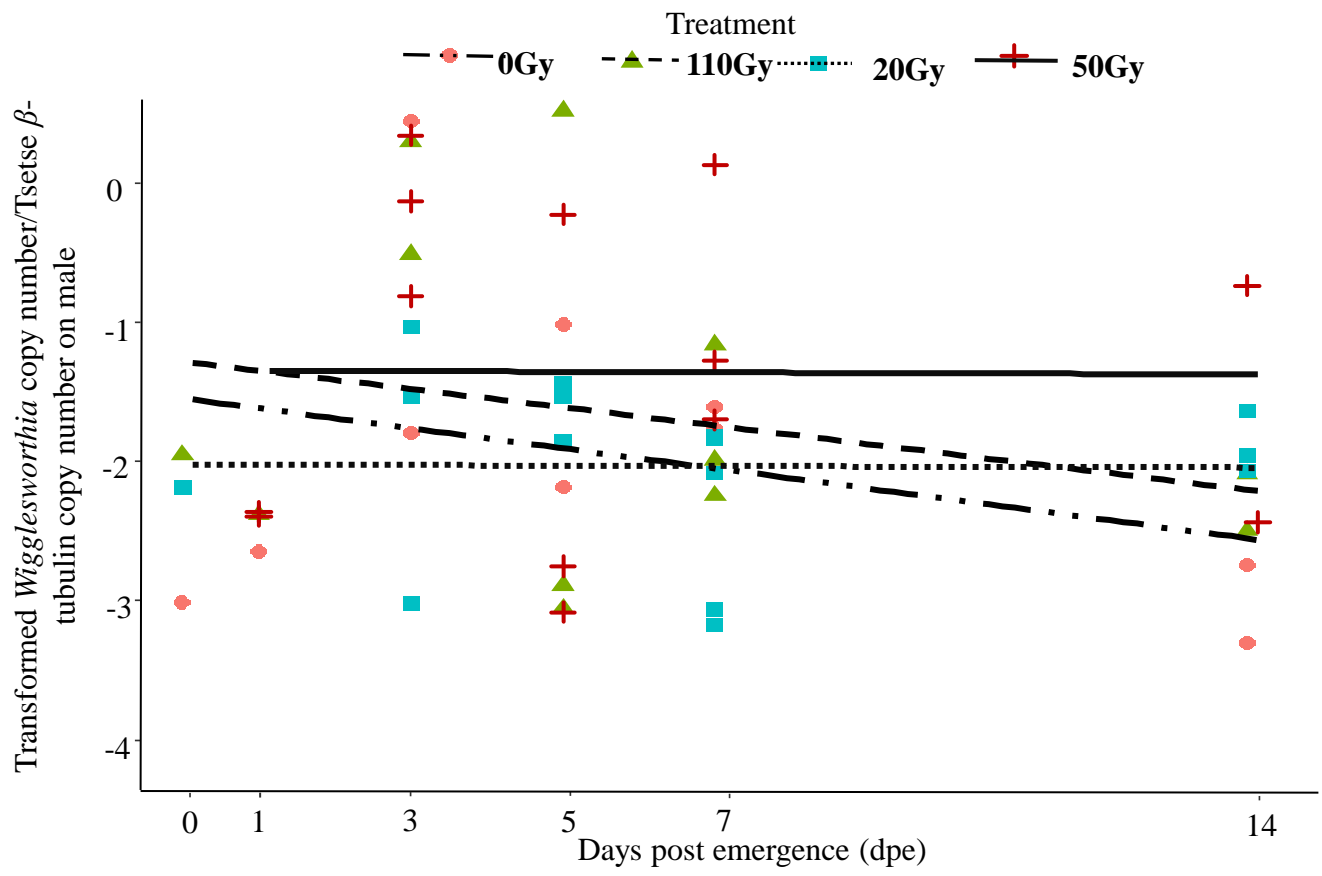**B**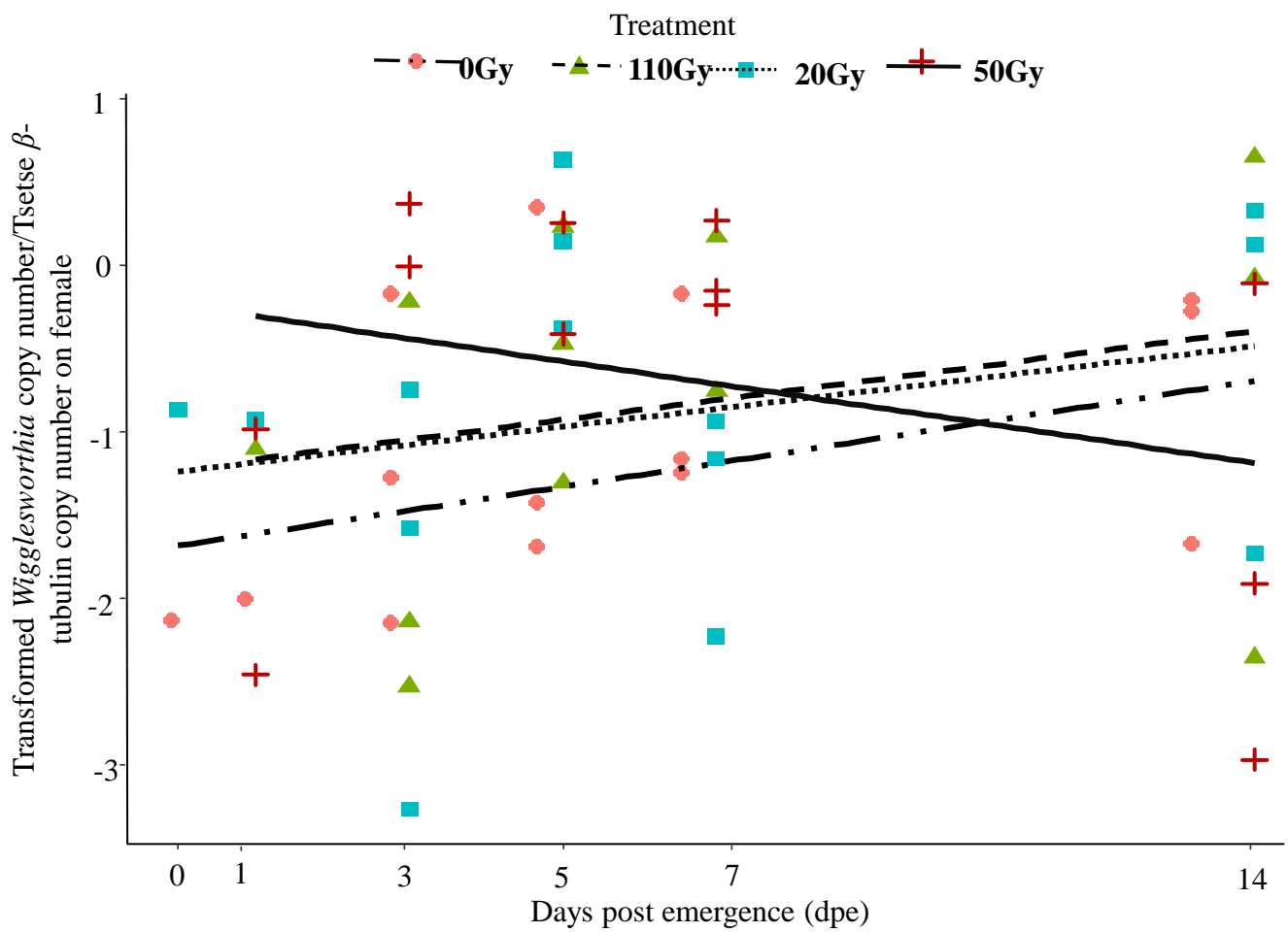

Supplement: Supplementary file 8 — Impact of time post irradiation on Wigglesworthia and Wolbachia copy number in G. m. morsitans adult flies emerged from irradiated 22-day old puparia. Four males of adults emerged from 22-day old puparia exposed to different radiation doses were used to quantify on Wigglesworthia male (A) and Wigglesworthia female (B) copy number at different time point post-irradiation for each irradiation dose. Normalized qPCR data were transformed (λ = 0.02 and λ = 0.3 for males and females respectively) to best fit the statistical normal distribution and used for the regression analysis. (PDF 99 kb) [file 12866_2018_1283_MOESM8_ESM.pdf]

**A**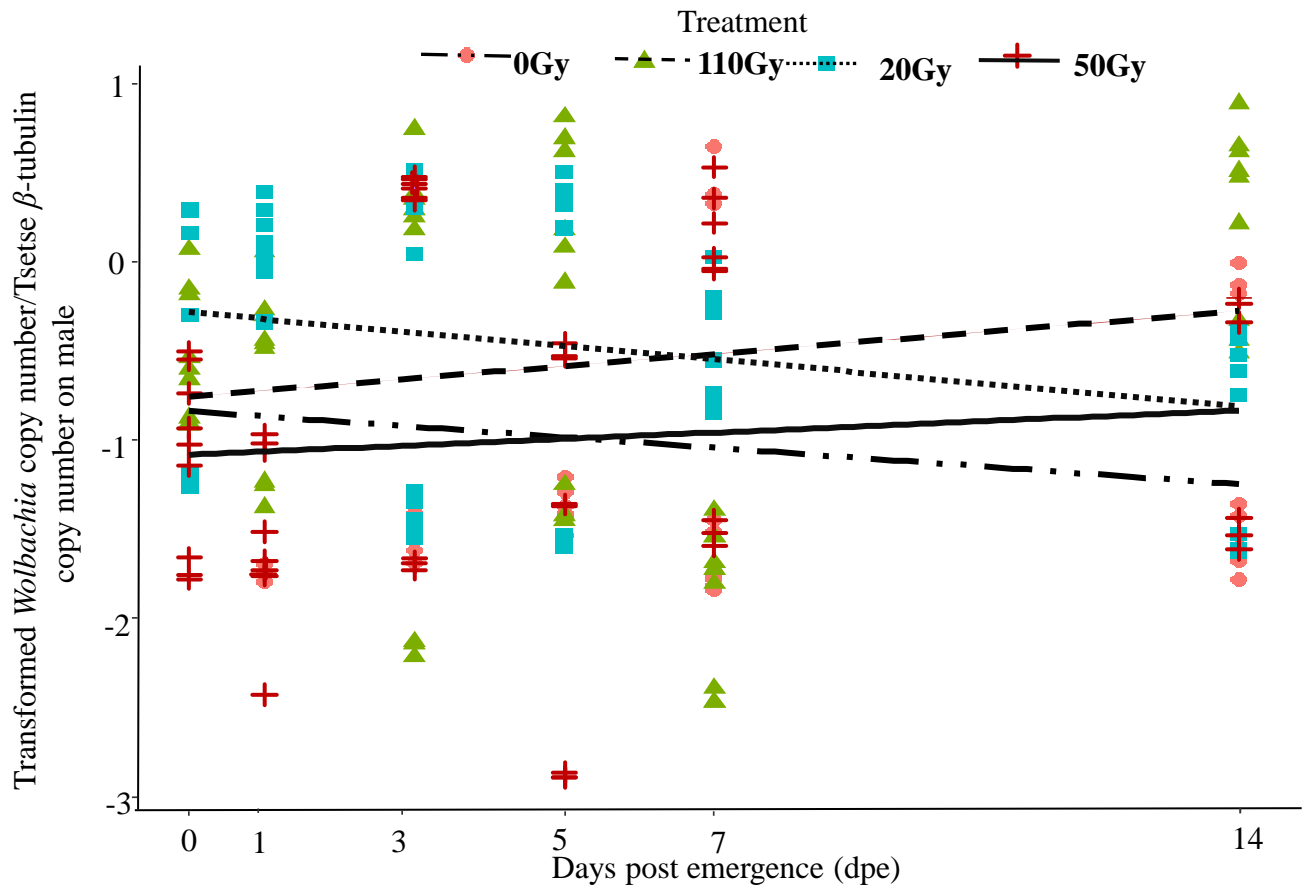**B**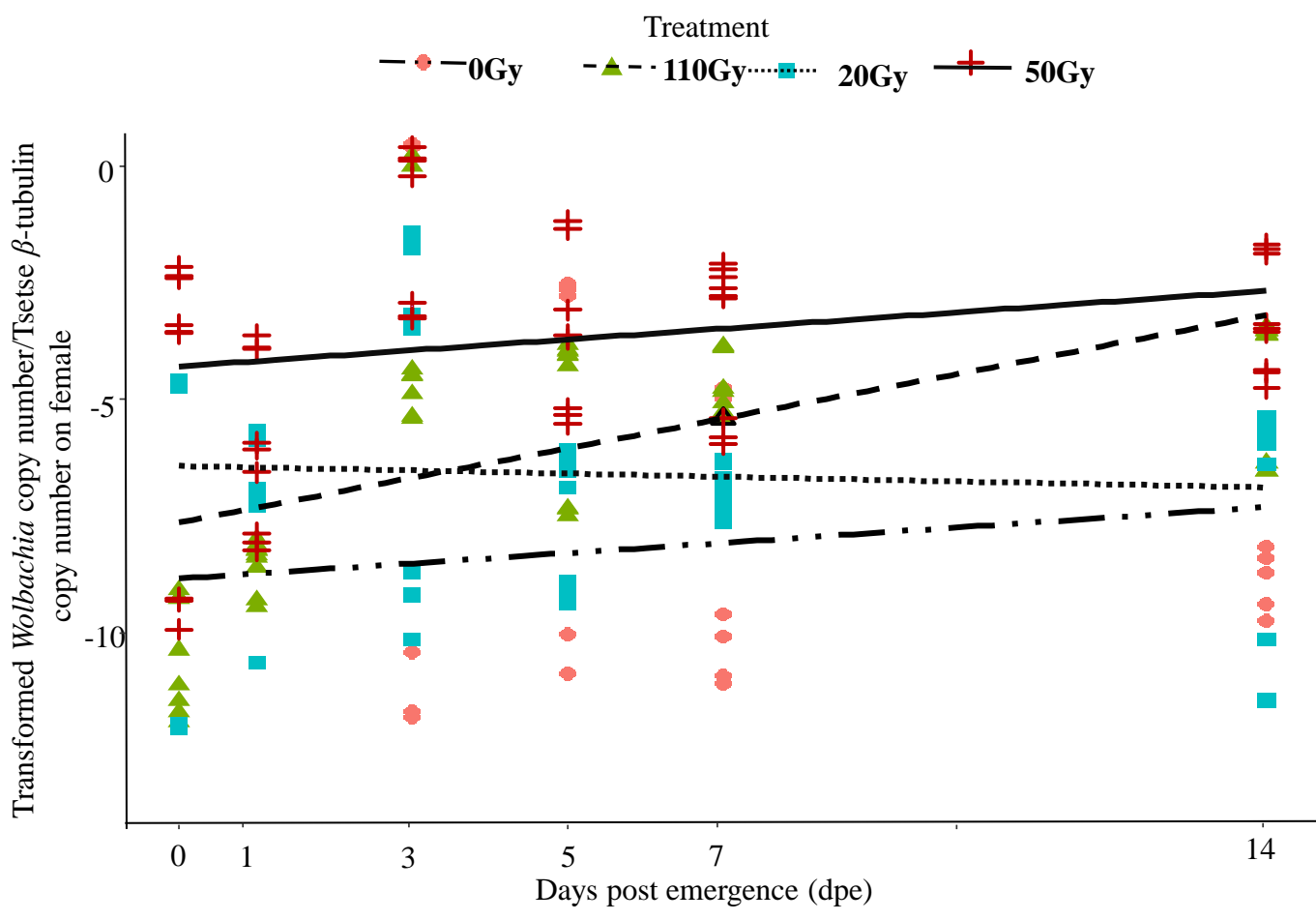

Supplement: Supplementary file 9 — Impact of time post irradiation on Wigglesworthia and Wolbachia copy number in G. m. morsitans adult flies emerged from irradiated 22-day old puparia. Four females of adults emerged from 22-day old puparia exposed to different radiation doses were used to quantify on Wolbachia male (A) and Wolbachia female (B) copy number at different time point post-irradiation for each irradiation dose. Normalized qPCR data were transformed ((λ = 0.2 and λ = − 0.04 for males and females respectively) to best fit the statistical normal distribution and used for the regression analysis. (PDF 127 kb) [file 12866_2018_1283_MOESM9_ESM.pdf]
